# Droplet digital microfluidic system for screening filamentous fungi based on enzymatic activity

**DOI:** 10.1038/s41378-022-00456-1

**Published:** 2022-11-21

**Authors:** Kenza Samlali, Chiara Leal Alves, Mara Jezernik, Steve C. C. Shih

**Affiliations:** 1 Department of Electrical and Computer Engineering, Concordia University, Montréal, QC Canada; 2 Centre for Applied Synthetic Biology, Concordia University, Montréal, QC Canada; 3 Department of Chemical Engineering and Applied Chemistry, University of Toronto, Toronto, ON Canada; 4 Department of Biology, Concordia University, Montréal, QC Canada

**Keywords:** Chemistry, Engineering

## Abstract

Fungal cell-wall-degrading enzymes have great utility in the agricultural and food industries. These cell-wall-degrading enzymes are known to have functions that can help defend against pathogenic organisms. The existing methods used to discover these enzymes are not well adapted to fungi culture and morphology, which prevents the proper evaluation of these enzymes. We report the first droplet-based microfluidic method capable of long-term incubation and low-voltage conditions to sort filamentous fungi inside nanoliter-sized droplets. The new method was characterized and validated in solid-phase media based on colloidal chitin such that the incubation of single spores in droplets was possible over multiple days (2–4 days) and could be sorted without droplet breakage. With long-term culture, we examined the activity of cell-wall-degrading enzymes produced by fungi during solid-state droplet fermentation using three highly sensitive fluorescein-based substrates. We also used the low-voltage droplet sorter to select clones with highly active cell-wall-degrading enzymes, such as chitinases, β-glucanases, and β-N-acetylgalactosaminidases, from a filamentous fungi droplet library that had been incubated for >4 days. The new system is portable, affordable for any laboratory, and user-friendly compared to classical droplet-based microfluidic systems. We propose that this system will be useful for the growing number of scientists interested in fungal microbiology who are seeking high-throughput methods to incubate and sort a large library of fungal cells.

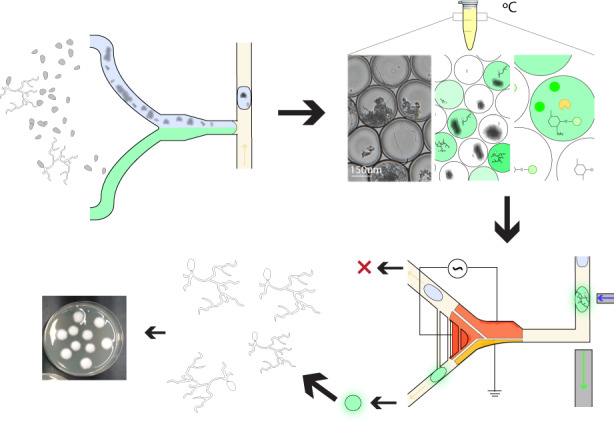

## Introduction

Filamentous fungi are often the preferred host for industrial biotechnology applications due to their natural ability to produce high yields of extracellular protein. For example, their proteins have been used for biomass hydrolytic degradation, pharmaceutical production, food industry ingredients, or agricultural fungicide applications^1–3^. *Clonostachys rosea* (also called *Gliocladium catenulatum*, teleomorph *Bionectria ochroleuca*) is a commonly studied fungal mycoparasite that is being used as an agricultural fungicide^4–6^. While the mechanisms are not fully known, the production of extracellular cell-wall-degrading enzymes is known to play a key role in *C. rosea*’s mycoparasitism^7^. Cell-wall-degrading enzymes of *C. rosea* include chitinases, glucanases, and proteases. The chitinases characterized in *C. rosea* mostly belong to the glycoside hydrolase (GH) family GH18, of which at least 14 genes were confirmed to be present in *C. rosea*, including different exo- and endo-chitinases^8^. β-1,3-, β-1,4-, and β-1,6-Glucanases make up the second group of cell-wall-hydrolyzing enzymes, of which β-1,3-Glucanase from *C. rosea* has been well characterized^9^. Due to its commercial application in organic agriculture, there is much interest in screening random mutant *C. rosea* libraries for improved field-relevant properties. Typically, filamentous fungi that produce active enzymes are obtained by implementing an efficient strain improvement strategy, which now frequently involves a high-throughput functional screen to select desired clones from mutant libraries^10,11^. There are many high-throughput systems available for bacteria and yeast; however, these are not well adapted to the filamentous fungal growth cycle or morphology. Fluorescence-activated cell sorting (FACS) and flow cytometry methods for filamentous fungi can only screen conidia and cannot be used to screen for cell-secreted products^12,13^.

Droplet microfluidics has become a popular method for high-throughput screening of single cells^14–17^. When cells are encapsulated together with fluorogenic components, single cells can be screened rapidly for enzyme activity with fluorescence-activated droplet sorting (FADS) procedures^18^ or visual-based feedback control^19^. These methods allow for activity analysis of secreted enzymes^20^, cell-bound proteins^21^, or intracellular protein products^16^. Zang et al. first showed that it is possible to incubate filamentous organisms (filamentous actinobacteria) in droplet-in-oil emulsions, followed by Mahler et al. and Tu et al.^22–24^. More recently, Beneyton et al. and He et al. demonstrated droplet incubation of filamentous fungi and the first use of FADS microfluidic systems for screening single conidia-derived filamentous fungi libraries based on secreted products^25,26^. However, there are two limiting factors that make the use of droplet microfluidics for filamentous fungi challenging. First, the droplet incubation time is limited by the growth rate of the organism. The fungi will start to form hyphae after spore germination, which will eventually pierce through the nano- or picoliter droplets after ~16 h of incubation, as reported for two fungi species *Trichoderma reesei* and *Aspergillus niger*^25,26^. As a result, droplets will often merge with neighboring droplets during incubation or are more likely to split or break during sorting, making it difficult to sort based on enzymatic activity. Second, when using dielectrophoretic (DEP) sorting, the system needs to be experimentally tuned according to droplet properties, such as size or conductivity. The long droplet incubation times needed for protein expression and the different individual growth rates contribute to droplet volume polydispersity post-incubation, which complicates the sorting procedure^25,26^. In addition, under high electric fields, the deformation of hyphae containing droplets will likely occur, risking the loss of the constituents from the droplets. Therefore, this two-fold challenge makes it difficult to screen filamentous fungi using standard droplet DEP-based systems, and new sorting systems that are adapted to the properties of filamentous fungi should be developed^27,28^.

Here, we describe the use of a droplet-based microfluidic system for high-throughput screening of enzymes with cell-wall-degrading activity in filamentous fungi. We introduce solutions to the challenges related to using droplet microfluidics for fungal culturing, sorting, and screening by optimizing the droplet incubation method and using a low-voltage-based sorting method. First, we cultured fungal spores in solid-state fermentation (SSF) media, such that hyphal growth was maintained in the droplet and incubation times could be prolonged. We incubated single spores of a *C. rosea* mutant library within droplets containing an enzymatic substrate and colloidal chitin-based solid-state media. We used three highly sensitive fluorescein-based substrates (FL-GlcNAc, FL-GalNAc, FD-Glc) that release fluorescein units during incubation to measure cell-wall-degrading enzyme activity. Prolonged incubation using SSF with colloidal chitin as a substrate was explored by evaluating the enzymatic activity of cell-wall-degrading enzymes in this media and comparing the activity to the liquid culture, and observing the droplet integrity over time. Finally, we integrated a low-voltage sorter (using an electrostatic-based sorting technique rather than dielectrophoresis^29–32^) tuned to the characteristics of filamentous fungi droplet libraries to seamlessly couple incubation and sorting. We explored the compatibility of the low-voltage sorter with a droplet microfluidic system to select clones with high cell-wall-degrading enzymatic activity, such as chitinases, β-glucanases, and β-N-acetylgalactosaminidases, from a long-term incubated (at least 4 days) filamentous fungi droplet library, which was further tested for their biocontrol ability and their prolonged activity. The addition of solid-phase culture in the droplets and low-voltage sorting is the first report, to our knowledge, to show a successful autonomous screening of filamentous fungi enzymatic activity several days after germination. The results presented here highlight the optimal use of droplet-digital microfluidics for fungal culturing and screening and illustrate how such a method can be useful for finding fungal proteins with applications in industrial biotechnology.

## Results and discussion

### Fungal screening system design and development

The isolation of mutants from a filamentous fungi library requires an efficient high-throughput screening strategy. While there are microfluidic enrichment strategies to sort out active fungi, they suffer from challenges related to droplet incubation and microfluidic sorting^25,26^. To solve these challenges, we introduce a filamentous fungi droplet screening system that consists of (1) a solid-state droplet fermentation (SSDF) incubation method and (2) a low-voltage droplet sorter suitable for maintaining the integrity of droplets containing filamentous fungi. Our electrostatic coplanar electrode sorter was optimized to sort fragile droplet libraries (i.e., the sorter did not burst the droplets containing fungi) and without the need to frequently tune the sorter parameters (i.e., applied voltage and flow rate) based on differences in droplet volume. Additionally, this system has a small footprint and does not require expensive components (e.g., microscopes, lasers, or specialized optics), making it more accessible to any user interested in screening fungal organisms or cell lines that are sensitive to high electric fields^27^.

**Fig. 1:**
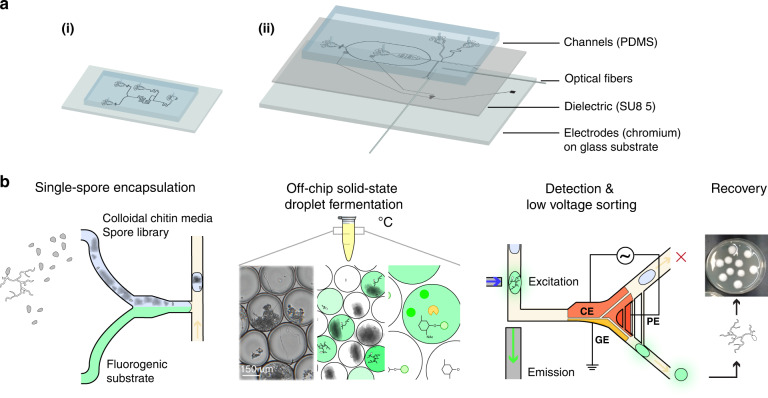
Microfluidic device to screen for cell-wall-degrading enzymes in filamentous fungi. **a** Schematic of two microfluidic devices used for screening: (i) a droplet generator and (ii) a sorting device containing electrodes. In (i) and (ii), the microfluidic channel is fabricated via soft lithography techniques except that (ii) contains an electrode layer with an SU-8 dielectric layer that is bonded to the channel PDMS layer. Two optical fibers (excitation: 105 μm core, 0.22 NA; emission: 200 μm core, 0.39 NA) are inserted into (ii) and placed orthogonally to the channel to excite and detect droplet fluorescence. **b** Screening workflow overview. Enzyme screening followed five steps. First, the mutant fungal population is generated through UV mutagenesis. Next, a microfluidic mixer T-junction droplet generator (see device (i)) co-encapsulates the conidial library suspended in colloidal chitin minimal media with a fluorescein-linked enzymatic substrate for glycoside hydrolases. The droplets were then collected and incubated in HFE7500 oil 2% fluorosurfactant at 27 °C for 2–4 days. The droplets contain single spores, minimal media with colloidal chitin, and a cell-wall-degrading specific fluorogenic substrate (FL-GlcNAc, FL-GalNAc, or FD-Glc). During incubation, glycoside hydrolase (GH) activity leads to the cleavage of the fluorescein-based substrate and the release of fluorescent fluorescein units that remain confined within the droplet. The droplet library is reinjected into the microfluidic low-voltage sorter (see device (ii)), which uses a three-electrode system (CE - constant electrode, PE - pulsing electrode, and GE - ground electrode). Mutant populations are sorted when they display high fluorescence intensity and are recovered on PDA plates and grown into clonal colonies, after which enzymatic assays are performed.

The system presented here (illustrated in Fig. 1a) consists of a droplet generator, and an electrostatic-based fluorescence-activated droplet sorter (shown in more detail in Supplementary Fig. 1). The droplet generator device is used to create the single-spore droplet library and consists of three inlets that are used for (1) the *C. rosea* spore solution in solid-state fermentation (SSF) media, (2) the fluorogenic enzymatic substrate solution, and (3) the continuous oil phase with 2% fluorosurfactant. The solutions from inlets (1) and (2) were mixed with a serpentine mixer, and droplets were generated via a T-junction mechanism. As illustrated in Fig. 1b, a spore library was exposed to UV irradiation to generate a mutant library and subsequently diluted in solid-state fermentation media (i.e., colloidal chitin in minimal media). In the generator, each component of the library was mixed with one of three highly sensitive fluorescein-based substrates (fluorescein mono-(*N*-acetyl-β-D-glucosaminide (FL-GlcNAc), fluorescein mono-(*N*-acetyl-β-D-galactosamine) (FL-GalNAc), or fluorescein-di-β-D-glucopyranoside (FD-Glc)), and a single spore was encapsulated in a droplet emulsion. Following droplet generation, each sample in the droplet library was incubated in a PCR tube at 26–36 °C in the dark for up to four days. The droplets were then injected into the sorter and sorted based on fluorescence. Within the droplets, the fluorescein substrates were cleaved by cell-wall-degrading enzymes, by which the activity of glycoside hydrolase (GH) family CAZymes (i.e., carbohydrate-active enzymes), such as N-acetyl hexosaminidases (e.g., chitinases) and β-glucanases, were measured^8,33^.

In the experiments described here, we explored the concept of using coplanar electrode sorters. Filamentous fungi expand their mycelium after several hours of growth and incubation, which causes the hyphal tips to exit the droplets. When the tips exit the droplets, they disrupt the droplet interface, and high-field DEP droplet sorting can lead to the bursting of the droplets and loss of the active enzymes during sorting^26^. Previously, electrostatic-based sorters have shown promising results and reliable droplet sorting without droplet breakage or damage^29^. This technique is based on applying kHz frequency potentials to electrodes under a dielectric layer and using the generated uniform electrostatic field to move the droplets. A binary sorter was designed (motivated by our previous work^31,32^ and others^29,30^) consisting of a droplet receiving inlet chamber followed by a spacer oil channel and a sorting junction. Three coplanar electrodes were placed under a Y-junction with the dual purpose of actively sorting positive (P) droplets toward the higher resistance (narrower and longer) channel and maintaining the preference of the negative (N) droplets toward the main (wider) channel (Supplementary Fig. 1C). The electrodes were configured as a constant electrode (CE) located at the top, a pulsing electrode (PE) located in the middle, and a ground electrode (GE) located at the bottom of the channel. The sorting mechanism is based on the formation of a uniform electric field between the gaps of the electrodes that depends on which electrodes are grounded (see Supplementary Fig. 2, Table 1 for simulation parameters). From our simulations, when a continuous potential (AC, 10 kHz) was applied to the CE (with PE + GE being grounded), an electric field was generated between the gap. This configuration ensures that N droplets are directed into the main channel. When a P droplet is detected, the PE is activated with a short and small pulse (300 ms, 10 kHz sine wave, 4.6–51.8 V_RMS_), such that an electric field forms along the gap between CE/PE and GE such that it directs P droplets toward the narrower channel (Supplementary Fig. 2).

Due to the limited number of examples of droplet microfluidic screening platforms for filamentous fungi and to improve accessibility for using droplet-based microfluidics (inspired by Cole et al. and Ahmadi et al.), we developed this system to have a reduced footprint, be more affordable and be user-intuitive compared to classical DEP-based FADS systems (Supplementary Table 2). In addition, we created a microfluidic device that will sort polydisperse droplets at similar throughput using lower electric fields compared to other fungal microfluidic sorters (Supplementary Table 3)^24,25,31,34^. Although we used optical methods to observe the droplets, this system does not use microscope optics for excitation or detection but instead uses optical fibers and relies on a single portable detector^31,34^. The operation of three electrodes under a low voltage opens up the possibility of further reducing the electronics to have a smaller footprint and significantly reducing the cost of the system. An overview of the software and hardware to operate the system and device is shown in Supplementary Information Note 1.

### Improving droplet incubation for filamentous fungi to analyze cell-wall-degrading enzymes

To perform an enzymatic assay on a microfluidic scale, single spores and substrates were encapsulated into nanoliter-sized droplets. These were then taken off-chip for incubation to allow for protein expression and secretion. After several hours of incubation, the droplets were reinjected into a device to be sorted for high activity^35–37^. When culturing filamentous fungi for enzyme production, longer incubation times (~days) are required since certain enzymes are not expressed until several hours after spore germination^25,26,33^. This problem was previously reported by Beneyton et al. and He et al.—the sorted positive droplets did not display the expected fold-increase improvement in enzymatic activity^25,26^. Since these single spores are confined to liquid bioreactors, culturing filamentous fungi in droplets is challenging because a balance is required between preventing hyphal exit and obtaining enzymatic production above the limit of detection. Previously, *A. niger* hyphal tips were shown to burst through 250 pL droplets at ~15 h post-incubation (hpi) and through 18 nL droplets at 32 hpi^26^. *T. reesei* was grown for up to 16 h, while the organism showed enzyme production at 24 hpi^25^. Working with larger droplets can increase incubation times, yet this requires systems with a lower limit of detection and adapted microfluidic sorters for larger volume droplets^26,37^. We addressed this issue by finding relevant sensitive fluorogenic substrates to allow for the early detection of enzymatic production and by adjusting the incubation media to prolong droplet incubation.

We hypothesized that SSF in droplets could potentially solve the incubation issue by maintaining fungi within the SSF substrate such that the solid media will limit hyphal tip exit. Previously, colloidal chitin was shown to be a good addition to liquid culture (for *C. rosea*^38^) and was used as a structural support^39^. In addition to providing support, colloidal chitin also plays a role in inducing cell-wall-degrading enzyme expression in mycoparasites, since chitin is a major fungal cell-wall component^38^. The use of gel-like or solid supports (microcarriers) in microfluidic water-in-oil droplets prolongs incubation times for mammalian cells^40^, yeast^41^ or bacterial colonies^42^, and filamentous fungi^43,44^. However, we believe this is the first demonstration of the use of a solid substrate for SSF of filamentous fungi within nanoliter water-in-oil droplets and the first report of the encapsulation of colloids for solid support.

**Fig. 2:**
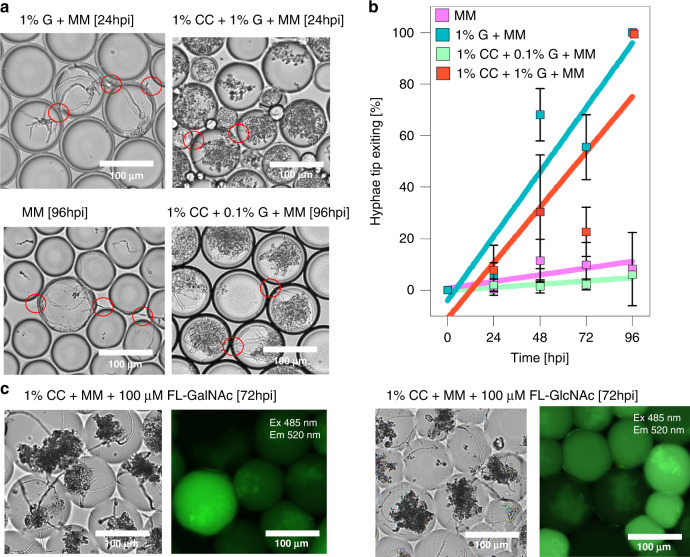
Incubation of filamentous fungi in droplets with varying media. **a** Images (40×) showing the morphology of *C. rosea* in droplets with varying media. For all droplets containing colloidal chitin (CC) with different concentrations of glucose (G), the filamentous fungi grew around the colloidal chitin, which acted as a structural support. Red circles indicate hyphae breaking through the droplets. **b** *C. rosea* hyphal tip exit from droplets during incubation in minimal media with 1% glucose (1% G + MM), minimal media with 1% glucose and 1% colloidal chitin (1% CC + 1% G + MM), minimal media with 0.1% glucose and 1% colloidal chitin (1% CC + 0.1% G + MM), and minimal media alone without any carbon source (MM). The percentage of hyphae exit was calculated by counting the number of droplets with hyphae exiting divided by the total number of droplets, including empty droplets, incubated for up to 96 h. **c** Solid-state droplet fermentation (SSDF) with cell-wall-degrading enzyme fluorescein-labeled substrate. Images (20x) showing droplets after 3 days of incubation with *C. rosea* spores germinating in colloidal chitin minimal media with FD-GalNAc. Fluorescein was maintained inside the droplet (no leakage was observed).

To perform SSDF, a mixing channel was used to mix 1:1 2% colloidal chitin solution with the substrate solution prior to droplet generation. To prevent clumping of the colloidal chitin and minimize clogging in the channel, the solution was filtered (10 µm), after which the spores were dissolved in the colloid solution with continuous stirring (with small stir bars) inside the syringe. Next, the solution was injected at a lower flow rate, which gradually increased to a flow rate equal to that of the substrate solution to avoid sudden clogging. With this method, we encapsulated the spores in four types of media: minimal media (MM; no carbon source), 1% glucose (G), and colloidal chitin (CC) with 0.1% or 1% glucose to determine whether the hyphal tips exited after incubation. As shown in Fig. 2a and Supplemental Fig. 3, hyphal tip exit occurred after 24 hpi, and droplet bursting was observed after 48 hpi in ~1 nL drops when 1% glucose was present. Interestingly, the addition of colloidal chitin showed hyphae clumps around the chitin inside the droplet (Fig. 2a) and reduced the rate of hyphal tip exit in 1% glucose and caused large variations in droplet volume (Fig. 2b for plots and Supplemental Tables 4 and 5 for rates and statistics), suggesting that colloidal chitin provides more structural support than a carbon source. Chitin as a structural support was also shown in other work, in which colloidal chitin was the only carbon source and favorably broke down the chitin rather than acting as a carbon source^38^. Moreover, colloidal chitin with a lower concentration of glucose (0.1%), the hyphal tip exit was only observed after four days (96 hpi in 1 nL droplets) and showed a hyphal exit rate similar to that in minimal media, further confirming the structural role of chitin. The effect of the carbon source in the droplet media on growth is limited, given that when droplets were incubated (27 °C, 2 days) in minimal media without any carbon source, we found several *C. rosea* spores germinating and developing hyphae. This can be explained by spore germination being mainly dependent on the presence of water and oxygen^45^, or *C. rosea* could have a mechanism to access the carbon chain of fluorinated surfactants in the HFE oil since the ability to degrade fluorinated compounds has been demonstrated for other fungi^46–48^.

Following culturing on colloidal chitin, we examined fluorogenic substrates that can be used to monitor the production of a collection of cell-wall-degrading enzymes from the glycoside hydrolase family^8,33^. Colloidal chitin has previously been used to enhance the production of chitinases and β-1,3-glucanases by *C. rosea*. We examined the production of cell-wall-degrading enzymes using three substrates, FD-Glc for β-glucosidases and β-1,3-glucanases, FL-GlcNAc for (exo-)chitinases, and FL-GalNAc for N-acetylgalactosaminidases. Previous reports have measured the enzymatic activity of cell-wall-degrading enzymes by endpoint assays that monitor the release of glucose units for β-1,3-glucanases or use 4-methylumbelliferyl substrates such as 4-methylumbelliferyl N-acetyl β-D-glucosamine (4-MU-GlcNAc for exo-chitinases)^38,49^. A preliminary well plate assay was performed to confirm enzymatic activity in the parent strain using 4-methylumbelliferyl β-D-glucopyranoside (4-MU-Glc), 4-MU-GlcNAc, and 4-methylumbelliferyl-N-acetyl galactosamine (Supplementary Fig. 4). All substrates showed stronger fluorescence after five days of incubation with *C. rosea* agar plugs (*P* < 0.05 compared to media without *C. rosea*). Increased fluorescence indicates that the parent strain natively produces enzymes that cleave the three enzymatic substrates. When we grew *C. rosea* in colloidal chitin minimal medium (CCMM), all three substrates showed significantly higher fluorescence in comparison to that observed for *C. rosea* grown in PDB (potato dextrose broth) medium (*P* < 0.05), indicating that CCMM upregulates cell-wall-degrading enzyme expression and that 4-MU-Glc is a poor choice to represent β-1,3-glucanase production. Next, the equivalent fluorescein-based substrates were used for a kinetic assay with spore solutions (first report to the best of our knowledge) to further investigate them as substrates for FADS. Common enzymatic substrates (e.g., 4-methylumbelliferone- and resorufin-based substrates) are difficult to use for detecting enzymatic activity in droplet microfluidic devices because they require merging at the endpoint or require UV excitation. Fluorescein-based substrates are extremely sensitive, easy to detect under 470 nm excitation, and have previously been used in droplet microfluidics with excellent droplet retention for long-term droplet incubation^20,50,51^. We qualitatively examined substrate partitioning and hyphal exit by incubating *C. rosea* spores germinating in droplets in colloidal chitin minimal media with FL-GalNAc and FL-GlcNAc for four days. Culturing the spores in the solid media showed no cross-contamination of fluorescein across the droplets and very minimal hyphal exit (Fig. 2c). Furthermore, we observed detectable fluorescence after the incubation of spores (0.5 × 10^7^ spores mL^−1^, Poisson spore concentration) with fluorescein substrates (Supplementary Fig. 5). The fluorescence signal using all three fluorescein-based substrates displayed an increasing trend over 16 h of incubation. The change in fluorescence over time for all three substrates was significantly different when comparing enzyme production in glucose-containing MM and CCMM (ANOVA, *p* > 0.05), indicating higher enzymatic activity in CCMM and higher sensitivity of the fluorogenic substrates. We also note that the N-acetylglucosaminidase and N-acetylgalactosaminidase substrates displayed stronger fluorescence intensity than the β-1,3-glucanase substrate. We expect FD-Glc hydrolysis to be an indicator for β-1,3-glucanase because this enzyme has previously been reported to hydrolyze other glucopyranoside-bound fluorogenic substrates^52–55^. However, previous reports have shown higher β-1,3-glucanase activity than chitinase activity, which we did not detect from the preliminary results above^8,38^.

### Optimization and characterization of the electrostatic sorter

Dielectrophoretic sorters often need to be experimentally tuned (potential, flow rate, and frequency) based on specific droplet volume and content for optimal sorting^26,37,50^. However, after incubating droplets for extended time periods, droplets often display a variation in volume, which is especially true for the long-term droplet incubation of filamentous fungi. Second, filamentous fungi hyphal tips are known to puncture droplets. Extensively changing the shape of the droplets through channel geometry or high-voltage DEP sorting (electro-splitting) can increase the risk of droplet breakage during sorting and post-incubation.

**Fig. 3:**
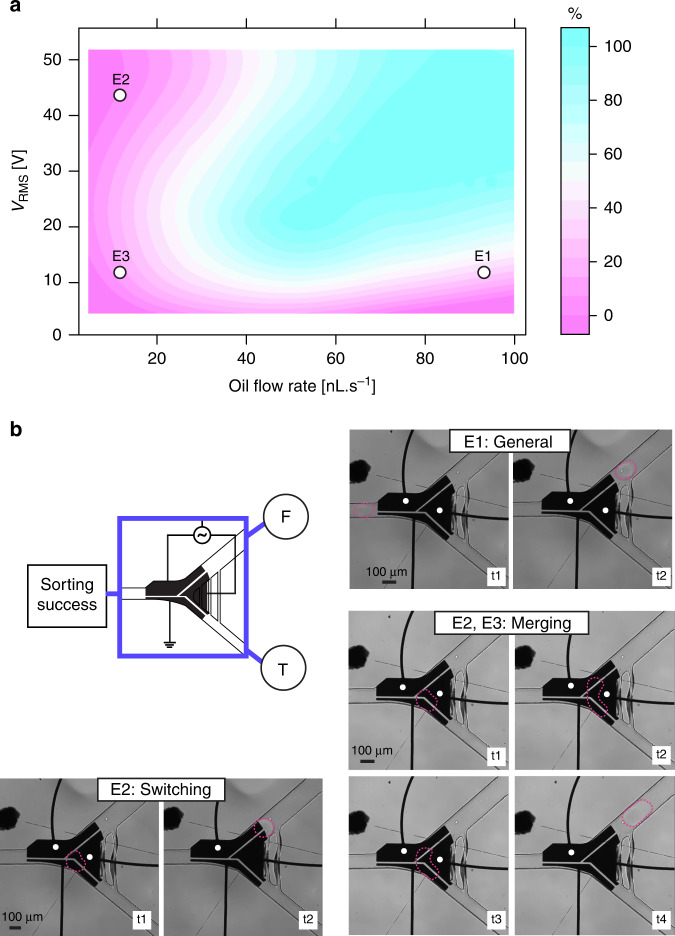
Evaluating the sorting efficiency at different flow rates and under different electric potential conditions. **a** A heatmap showing the efficiency of electrostatic droplet sorting (%) at different oil flow rates (nL·s^−1^) and applied potentials (V_RMS_). The success rate of sorting was determined by counting the number of droplets that successfully entered the disfavored channel (true, T) and those that entered the main channel (false, F). The highest sorting success is displayed as a blue area, and the lowest success is shown as the pink area. The optimal flow rate and potential to achieve 100% success are >51.5 nL·s^−1^, 27.4 V_RMS_. The lowest potential at which we observed perfect sorting (100%, *N* = 10) was at 12.5 V_RMS_, 50 nL s^−1^. This graph was created by polynomial fitting using a binomial regression with an interaction term, AIC: 1029.1 with coefficients *P* < 0.05, *N* = 10 per condition. Three failed sorting conditions were observed and labeled E1, E2, and E3. **b** Time-course snapshots of three failed sorting conditions. Actuated electrodes are indicated with a white dot, and the droplet is outlined in red. E1: Droplets immediately enter the main channel (low potential, high flow rate). E2: Droplets enter the disfavored channel, but switch to the main channel after the PE pulse (low flow rate; high potential). E2 and E3: Droplets can merge with the next arriving droplet (low flow rate)

We first tested the effect of changing the flow rate and potential on droplet emulsions of identical size and content (~1 nL, ddH_2_O). The experimental results showed that the sorting was successful (number of droplets that entered the disfavored channel (T) divided by the total (T + F)) and modeled with a binomial regression model with an interaction term (all terms *P* < 0.01). A potential was sent to the top constant electrode (CE) while grounding the other two electrodes (PE and GE). For each condition, sorting was performed manually by applying a potential pulse to the PE upon the arrival of a droplet (300 ms; *N* = 10) to sort droplets at random. A sine wave of 10 kHz with varying AC potential was amplified to obtain a potential between 4.6 and 51.8 V_RMS_ (see linear calibration curve Supplementary Fig. 6), and the flow rates of the spacer oil were varied between 10 and 100 nL s^−1^. As shown in Fig. 3a, reliable efficient sorting occurred at oil flow rates greater than 51.5 nL·s^−1^ at an applied voltage of 27.4 V_RMS_ (*P* = 0.5, binomial regression inflection point). The lowest potential at which we observed perfect sorting (100%, *N* = 10) was at 12.5 V_RMS_, with a flow rate of ~50 nL·s^−1^ (Supplementary Fig. 7 for dot plot). Electrostatic-based sorting has been reported previously^29,31^, but this is the first example of the characterization of a low-voltage sorting system with perfect fidelity. While the sorter performed efficiently in this region (shown in blue), we note that there are three regions (labeled E1, E2, and E3) where sorting errors occurred more frequently. E1 occurs when droplets enter the main channel. This frequently happens when sorting at low voltages (<27.4 V_RMS_), with higher flow rates (>51.5 nL·s^−1^), and where the hydrodynamic drag force is larger than the electrostatic force. The second scenario (E2; Fig. 3b) occurs at low flow rates (<51.5 nL·s^−1^). Here, sorting fails because the hydrodynamic drag force is not strong enough to overcome the channel resistance in the disfavored channel, and the droplet either moves toward the main channel or remains static at the entry of the disfavored channel on the gap between the grounded and activated electrode due to a strong electrostatic field until the PE is turned off. In addition, when a second droplet arrives at the sorter junction when the PE is still on, merging can occur at high potentials (>27.4 V_RMS_) (E3; Fig. 3b). We hypothesize that when the orthogonal hydrodynamic drag forces and electrostatic forces (from the applied potentials) balance each other, sorting will be successful. By using this coplanar electrode configuration with an electrode gap oriented in parallel with the flow streamlines, electrostatic force-based sorting can be performed successfully at significantly lower applied voltages compared to dielectrophoretic techniques requiring up to 1.4 kV without sending potentials through the droplet content^26,29,50^. This can reduce deformation and the risk of droplet breakage, which is beneficial when working with fragile filamentous fungi in droplet libraries.

Furthermore, we tested the sorting efficiency for polydisperse droplet emulsions under two different sorting regimes (60 nL·s^−1^ or 80 nL·s^−1^). The droplet emulsion represents a reinjected droplet population of long-term droplet-incubated filamentous fungi. We observed droplets of different volumes passing through the binary sorter at two flow rates (60 nL·s^−1^ or 80 nL·s^−1^) without actuating any electrodes (Supplemental Fig. 8A). At 80 nL·s^−1^, the probability *p* of a droplet entering the main channel (labeled as ‘F’) is greater than 99.4%, while at 60 nL·s^−1^, the probability is greater than 98.9% (99.9% confidence level), suggesting that <1% of the true or false-positive droplets could be due to the channel geometry (Supplemental Fig. 8B). We also observed that at flow rates lower than 10 nL·s^−1^ and with increasing droplet size, the droplet plugs increased the resistance in the main channel, resulting in droplets frequently entering the disfavored channel. Next, we used the previously described manual electrostatic sorting method in two experiments with different sorting regimes (36.34 V_RMS_, 60 nL·s^−1^ and 44.20 V_RMS_, 80 nL·s^−1^) to assess the ability of an electrostatic sorter to sort varying droplet sizes under the same sorting conditions (Supplementary Fig. 8C). Droplets with varying volumes smaller than ~1 nL (at success >0.5; inflection point) could be sorted successfully under the same flow rate (60–80 nL·s^−1^) and potential (36.34–44.20 V_RMS_). This indicates the ability to sort polydisperse solutions without the need for retuning the sorting parameters, such as voltage, pulse length, or oil flow rate, for different volumes^56^. This is beneficial to sort filamentous fungi-containing droplets, which can often merge or display polydispersity after long-term incubation due to different growth rates or evaporation.

**Fig. 4:**
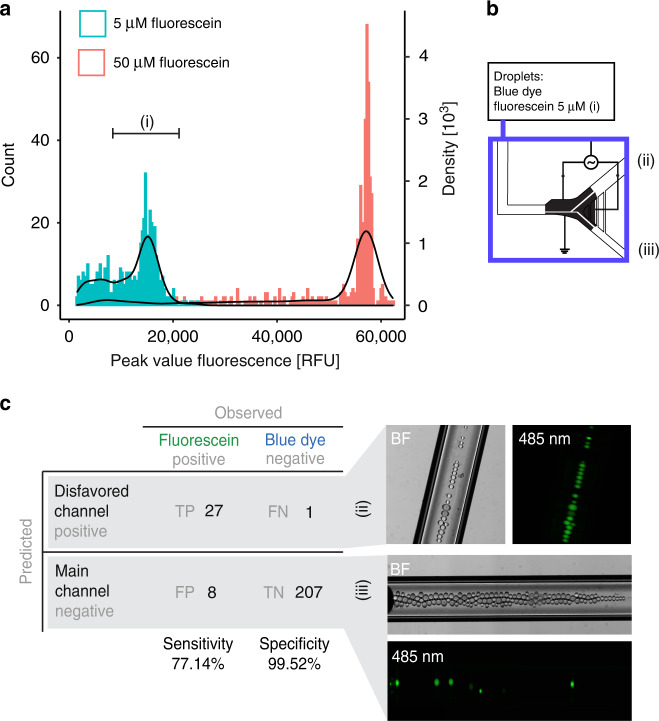
Calibration of the low-voltage sorter. **a** Gating histogram. Peak intensities of the 5 µM (shown in blue) and 50 µM (shown in red) fluorescein-containing droplets were measured when passing through the microfluidic sorter using a peak detection algorithm (*N* = 500). **b** Experimental overview. A mixed droplet library was reinjected into the sorter, of which the sorting gate was set to fit the 5 µM fluorescein histogram, and autonomous sorting with total oil flow of 0.03 µL/s, PE 10 kHz, 15 V_RMS_, 0.3 s, and droplet travel time of 0.1 s. **c** Droplet populations from each outlet after autonomous sorting of a mixed population of 5 µM fluorescein positive (P) droplets and negative (N) blue dye droplets. Sorted positive droplets are expected to flow into the disfavored channel, with the negative droplets flowing into the main channel. From observation of the bright-field and 480 nm-excited fluorescence microscopy images of the droplets recovered from both sorting outlets, the sensitivity (77.14%) and specificity (99.52%) of the sorter were calculated.

After manually characterizing the successful sorting regimes to sort polydisperse droplet emulsions, we analyzed the performance of fully autonomous sorting under a single hardware configuration. All sorting manipulations were controlled using our automated system (previously published^32^) with additional developed software for droplet fluorescence detection and automated electrode pulsing, including a graphical user interface. We evaluated several performance measures, such as the sensitivity, specificity, and throughput of sorting when automatically detecting and sorting positive droplets containing 5 µM fluorescein. A droplet-generating device containing two independent T-junction droplet generators was used to produce predefined droplet libraries for sorter calibration with fluorescein standard droplets (5 µM and 50 µM), and ddH_2_O droplets dyed blue (0 µM fluorescein) (device 2; Supplementary Fig. 1). As a first step, to set the sorting gate, we measured the fluorescence of droplets with different concentrations of fluorescein with a peak finding algorithm (Fig. 4a and Supplementary Note 2). We generated droplets with 5 µM and 50 µM fluorescein, reinjected the droplets into the sorter, and determined the fluorescence intensity range for the 5 µM fluorescein droplets. Next, a mixed population of 5 µM fluorescein droplets with blue-dyed droplets (representing 0 µM fluorescein) was generated and reinjected into the sorter. Using the previously determined 5 µM fluorescein intensity range as a gate, a sorting voltage of 15.8 V_RMS_, 10 kHz sine, and a flow rate of 30 nL·s^−1^, the device was left to sort autonomously. Populations of fluorescein droplets and blue dye droplets were collected from each outlet in capillaries and observed after autonomous sorting (Fig. 4b). The results are shown in a confusion matrix (Fig. 4c, Supplementary Table 6), displaying a sensitivity of 77.1% (true positives out of the total number of fluorescein droplets) and specificity of 99.2% (true negatives out of the total number of blue-dyed droplets). The sensitivity is lower than that of other reported sorters^50,57^, but it can be further increased by more precise timing of PE actuation, given that our system relies on experimental (and not in situ) measurements of droplet speed and derived droplet travel time to actuate the electrodes for sorting (Supplementary Fig. 9). Although our sorting throughput of 7 Hz is slightly lower than that of other reported sorters^37,50,57^, changing the system to a laser- and PMT-based detection system, the electrode configuration, the channel geometry or optimizing communication speed in the electronics will increase the throughput of our sorting system to the kHz range^29,37^. Comparing our system to current filamentous fungi screening techniques, 7 Hz is faster than standard high-throughput well plate-based methods and comparable to the previously reported microfluidic throughput for fungal sorting (Beneyton et al. (10 Hz))^26^. To summarize, we developed an electrostatic sorter that can efficiently sort polydisperse volume droplet libraries at 7 Hz throughput and with an applied potential of only 12.5 V_RMS_ without exposing the droplet contents to an electric field and risking electro-splitting. This new sorter is an important contribution that can aid in the automated sorting of filamentous fungi droplet libraries.

### Application: screening glycoside hydrolases in *C. rosea* using droplet-based chitin fermentation

Mycoparasitism by degrading pathogenic fungal cell walls (nercophytism) is a crucial mechanism of filamentous fungi that are used as biocontrol agents^58^. To show the use of our system, we generated a random mutant *C. rosea* conidia library and screened single clones for improved secreted cell-wall-degrading enzymes. The generated UV mutant library showed an average spore survival of 11.5% compared to an untreated sample.

**Fig. 5:**
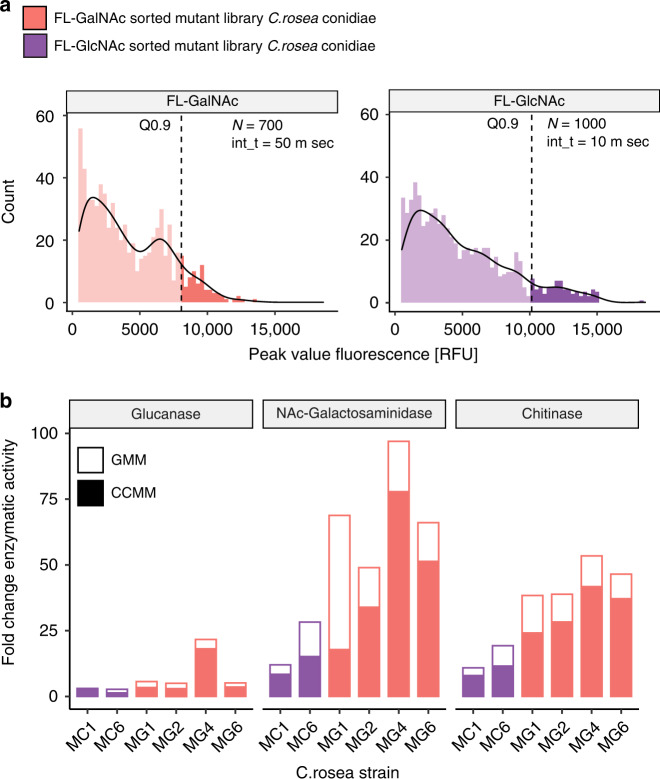
Sorting and recovery of C. rosea mutants based on the activity of cell-wall-degrading enzymes. **a** Peak intensity histogram and enzymatic assays using FL-GlcNAc (right) and FL-GalNAc (left) as the substrate for single-spore libraries. For the histogram, the fluorescence intensity of each droplet was measured in a mutant population after incubation at 27 °C. The intensity of the peaks between the wavelengths 510 nm and 520 nm were used for gating. To sort droplets, the intensity gate was set at the 0.9 quantile of the mutant population fluorescence (shown by the dotted line). The total number of peaks (N) and integration time (int_t) are indicated on the plot. The top 10% of the droplets were recovered on plates and cultured to obtain distinct mutant colonies (*MG* for the FL-GalNAc-sorted spores, *MC* for the FL-GlcNAc-sorted spores). **b** Three cell-wall-degrading enzymes were assayed to determine the top active mutant strains using 4-MU-GlcNAc (indicating chitinase activity), 4-MU-GalNAc (indicating N-acetylgalactosaminidase activity), or 4-MU-Glc (indicating β-1,3-glucanase activity). Fold change values were obtained with an endpoint enzymatic assay (pH 5.1, 30 min) and normalized to the activity of the wild-type (*N* = 3).

To screen the mutants for three different cell-wall-degrading enzymes, the fluorescein substrate for each enzyme was co-encapsulated with 2× CCMM with spores (1:1 microfluidic mixing). The resulting ~1 nL droplets were subsequently incubated at 27 °C for varying lengths of time (2–4 days) depending on the substrate. Before active sorting, we measured the fluorescence of each droplet of the reinjected droplet population to assess the differing fluorescence between the wild-type and mutant libraries and differences in the fluorescence profiles between substrates (Supplementary Fig. 10). The skewed populations (Pearson coefficient, mutant populations skewed right compared to the wild*-*type) confirmed that a majority of the droplets were empty (Poisson distribution encapsulation), displaying the background fluorescence of the substrates in empty droplets. FD-Glc-containing droplets showed a high degree of breakage after 2 days, and it was difficult to obtain intact droplets for sorting. Large clusters of hyphae were found, indicating droplet merging events and faster growth rates. As a result of the shorter incubation time, the peak histogram of the parent strain population could not be distinguished from the mutant population (Supplementary Fig. 10). While we confirmed that *C. rosea* generates β-1,3-glucanases in CCMM, we observed from endpoint and kinetic assays that the production was much lower than expected compared to the previous reports^8,38^. We hypothesize that *C. rosea* produces exo-glycosidases and exo-glucosidases in addition to endo-glycosidases, which can lead to the hydrolysis of FD-Glc, the release of glucose, and expedited growth rates and droplet breakage. Due to the failed attempt to sort based on FD-Glc, we proceeded with autonomous sorting of the FL-GlcNAc (one sorting experiment) and FL-GalNAc populations (two sorting experiments) (Fig. 5a). For FL-GlcNAc- and GalNAc-based sorting, the droplets were incubated for 2 and 4 days, respectively, after which the spore-containing droplets showed high levels of fluorescence that was distinguishable from empty droplets. For both substrates, the histogram displayed a difference in variance between the parent strain and mutant library (Levene test, *P* < 0.05), which confirmed the droplets with higher fluorescence in the mutant library. In a 30 min experiment, we screened, on average ~12,500 droplets at 7 Hz, which resulted in the screening of ~3800 unique single spores. The sorting gate was set to sort droplets for three different enzymes above the 90% quantile of the mutant populations, with 500 < λ_em_ < 520. Using the sorting regime as previously optimized (~40 V_RMS_, 60 nL·s^−1^ oil flow), positive droplets with high-yield GH production were collected in a glass capillary and spread onto potato dextrose agar (PDA) for verification. Over fifty colonies were randomly selected and subjected to further enzymatic assays. From the microfluidic screen, 13 strains from FL-GalNAc-based screen (*MG* strains) and 7 strains from the FL-GlcNAc-based screen (*MC* strains) were studied using a 4-MU substrate endpoint assay.

To evaluate prolonged enzyme activity, mutation persistence, and expression, we measured the specific enzymatic activity of the recovered mutants after three generations of culture over four days in both GMM and CCMM (Supplementary Fig. 11). The rates of all the strains were measured after four days in glucose (GMM) or glucose and chitin media (CCMM) (Supplemental Fig. 12). In CCMM, two MC strains (MC1 and MC6) and four MG strains (MG1, MG2, MG4, and MG6) showed the highest rates among all three enzymes (see Supplemental Tables 7–9 for the enzymatic activity of each strain grown in GMM and see Supplemental Tables 10–12 for the enzymatic activity of each strain grown in CCMM), with an average 1.9-, 19.4-, and 9.4-fold improvement in glucanase, galactosaminidase, and chitinase activity, respectively, over the wild-type strain (Fig. 5b). Although we screened the mutants in chitin, we observed the best improvements (5.28-, 34.0-, and 25.1-fold increases in glucanase, galactosaminidase, and chitinase activity, respectively) when these strains were assayed in glucose minimal medium. Indeed, future work is needed to understand these increases; however, these results indicate that sorting based on endpoint fluorescence measurements using our method of solid-state incubation and low-voltage sorting are selective for the most active cell-wall-degrading enzymes.

## Materials and methods

### Reagents and materials

Materials for the fabrication of the microfluidic devices include a transparent photomask (CAD/Art Services Inc., Bandon, OR), S1811 positive photoresist coated glass slides (Telic, Valencia, CA, USA), an MF321 developer (Rohm and Haas, Marlborough, MA, USA), a CR-4 chromium etchant (OM Group, Cleveland, OH, USA), an AZ-300T photoresist stripper (AZ Electronic Materials, Somerville, NJ, USA), <100> Si wafers (Silicon Valley Microelectronics Inc., Santa Clara, CA, USA), indium tin oxide-coated glass (Delta Technologies, Loveland, CO), and SU-8 5, SU-8 2075, and SU-8 developers (Microchem, Westborough, MA, USA). Optical fibers and bandpass filters were obtained from Thorlabs (Newton, NJ, USA). Polydimethylsiloxane (PDMS, 184 Sylgard) was purchased from Dow (Toronto, ON, CA), and chlorotrimethylsilane was purchased from Sigma‒Aldrich (Oakville, ON, CA). The polylactic acid (PLA) material for 3D printing was purchased from Shop3D (Mississauga, ON, Canada). DI water had a resistivity of 15 MΩ·cm^−1^.

Reagents for device operation included 3 M Novec HFE7500 engineering fluid and the surfactant 3 M Novec 1720 (M.G. Chemicals, Burlington, ON, CA), and PEG fluorosurfactant dissolved in HFE7500 (20 g of 5% wt) (Ran Biotechnologies, Beverly, MA, USA). All liquids were filtered with a Nylon filter cartridge (0.22 µm, Millex^®^GP, Millipore) prior to use. All glass syringes were from Hamilton (Reno, NV, USA). All tubing and fittings were sourced from IDEX Health & Science LLC (Oak Harbor, WA).

Fluorescein- and 4-methylumbelliferyl-based fluorescent substrates, chitin, the Bradford assay reagent, and all other cell culture and assay reagents were acquired from Sigma‒Aldrich (Mississauga, Canada) unless specified otherwise.

### Microorganisms and culture conditions

*Clonostachys rosea* cultures were obtained from a confidential source. *Fusarium graminearum* DAOMC 215630 and *Botrytis cinerea* DAOMC 143576 were obtained from the Canadian Collection of Fungal Cultures (Ottawa, CA). All fungi were cultured on potato dextrose agar (PDA) or in potato dextrose broth (PDB) supplemented with 0.05 g·L^−1^ chloramphenicol at 27 °C with 12 h light/dark cycles. For *Clonostachys rosea* cultures, after ~3 weeks, dark green aerial conidia formed. The conidia were harvested by washing each culture with sterile 0.01% Tween-80 in ddH_2_O, filtering the conidia through a 10 µm filter (Pluriselect, CA, US), centrifugation (8000 rpm, 2 min) and resuspension in the Tween-80 solution. The spore stock solution concentration was determined with a hemocytometer and was kept at 4 °C for up to 2 weeks until further use. The mutant conidia library was generated by UV mutagenesis. Ten milliliters of a 5 × 10^7^ spores·mL^−1^ suspension was spread on a sterile petri dish and treated with UV light (254 nm, 100 mJ/cm^2^) (UVP HL-2000 Hybrilinker cross-linker). Suspensions were kept in the dark, and a small aliquot was taken, serially diluted in sterile water, and spread on PDA plates to evaluate the number of viable spores. The remainder of the UV-treated suspension was plated on PDA and germinated at room temperature for 10–14 days. Aerial spores were collected, and the library was finally stored at −80 °C in 25% glycerol.

### Enzyme production media

Minimal media (MM) was used as the base media for incubation in droplets and was adapted from Mania et al. (2010) with an EDTA-based Hutners Trace element solution and 0.05% chloramphenicol^59^. MM was supplemented with either glucose or colloidal chitin (1% w/v) as the carbon source. Cell-wall-degrading enzyme production by *C. rosea* was induced by culture in liquid MM without glucose and 1% w/v colloidal chitin as the carbon source. Colloidal chitin also served as solid-state fermentation support. Colloidal chitin for enzymatic assays and droplet-based assays was produced following Wu, Cheng, and Li’s method, dissolving chitin (enzymatic assay grade) in 1 M HCl overnight at 40 °C^60^. The solution was centrifuged and neutralized by washing it with distilled water until a pH of 2–4 was reached. The final wash was performed with 2× MM, and colloidal chitin was formulated into a 2% w/v solution in 2× MM. Colloidal chitin for the biocontrol assay was produced following the Subramanian et al. method by dissolving chitin (practical grade) 1:10 in HCl (1 M) with stirring followed by incubation overnight at 40 °C^61^. The colloidal chitin was precipitated by slowly adding five volumes of chilled ethanol with constant stirring at 4 °C. The solution was centrifuged and neutralized by washing it with sterile distilled water and adding sodium acetate. The supernatant was discarded, the colloidal chitin pellets were dried, and 1% w/v colloidal chitin in MM was formulated for the biocontrol assay.

### Well plate enzymatic assays

For the endpoint enzymatic assay, PDB was inoculated with a 5 × 5 mm mycelium stab from one-week-old cultures and maintained in static culture at 27 °C for five days in a 2 mL 96-well deep well plate. For prolonged enzymatic assays, CCMM and GMM were inoculated with a 7 mm diameter mycelium stab from 2-week-old cultures (grown for two generations) and maintained agitated at 170 rpm at room temperature for 4 days in a 15 mL Falcon tube. Cell-free enzyme-containing supernatant was collected by centrifugation (4000 rpm, 4 min) and filter sterilization (0.22 µm). The supernatant was kept at 4 °C until use. Three 4-methylumbelliferyl enzymatic substrates, 4-methylumbelliferyl-N-acetyl-β-D-glucosaminide (4-MU-GlcNAc), 4-methylumbelliferyl-N-acetyl-β-D-galactosamine (4-MU-GalNAc) (BioSynth Carbosynth, UK), and 4-methylumbelliferyl-β-D-glucopyranoside (4-MU-Glc) (BioSynth Carbosynth, UK), were dissolved to generate 500 mM stock solutions in DMSO. A 50 mg mL^−1^ 4-MU standard stock solution was prepared in methanol. For the endpoint assay performed on mutant or *wt C. rosea*, each well of a standard clear 96-well plate was loaded with the substrate at a final concentration of 0.5 mM in sodium acetate buffer (pH 5.1) (75 µL) and the sample supernatant or standard (1.9 nmol·mL^−1^) (25 µL) for 30 min of incubation at 37 °C. Stop solution (100 µL) composed of sodium carbonate (0.4 M) was added, and the relative fluorescence was measured at *λ*_ex_ = 360 ± 20/λ_em_ = 450 ± 30 nm (40 flashes per well, 200 rpm orbital shaking before each measurement) in a fluorescence microplate reader (ClarioSTAR®, BMG Labtech). Background fluorescence was subtracted. To calculate enzymatic activity (U mL^−1^), the following equation was used:

$${{{\mathrm{Enzymatic}}}}\,{{{\mathrm{activity}}}}\left(\frac{{{{\mathrm{U}}}}}{{{{{\mathrm{ml}}}}}}\right) = \frac{{1.9 \times V_F \times {\mathrm{DF}} \times \left( {{\mathrm{FLU}} - {\mathrm{FLU}}_{{\mathrm{blank}}}} \right)}}{{{\mathrm{FLU}}_{{\mathrm{std}}} \times t \times V_{{\mathrm{sample}}}}},$$

where FLU is the fluorescence of the well [RFU], FLU_blank_ is the fluorescence of the substrate working solution [RFU], V_F_ is the final reaction volume [mL], DF is the enzyme dilution factor, FLU_std_ is the fluorescence of the standard solution minus the fluorescence of the standard blank, t is the incubation time of 30 min [min], and V_sample_ is the volume of the sample in the well [mL]. One unit of enzymatic activity will release 1 μmole of 4-MU from the appropriate substrate per minute at pH 5.0 and 37 °C.

For a kinetic enzymatic assay representative of droplet incubation, CCMM or MM (1% w/v glucose) was inoculated with 0.5 × 10^7^ spores mL^−1^ wild-type *C. rosea* per well. Three fluorogenic substrates, fluorescein-N-acetyl-β-D-glucosaminidase (FL-GlcNAc) (Abcam, Waltham, MA, USA), fluorescein-N-acetyl-β-D-galactosaminidase (FL-GalNAc) and fluorescein-di-glucopyranoside (FD-Glc), were dissolved in DMSO to generate stock solutions with concentrations of 100 mM. The substrates were dissolved in ddH_2_O and mixed with the samples to a final concentration of 100 µM. The assay was carried out overnight (16 h) in a 50 µL half-area flat bottom dark well plate (Greiner Bio-One, AT) incubated at 27 °C in a fluorescence plate reader (ClarioSTAR®, BMG Labtech), and measurements were acquired at λ_ex_ = 485 ± 15/λ_em_ = 530 ± 10 nm (100 flashes per well, 200 rpm orbital shaking before each measurement).

### Device fabrication and optical fiber setup

The microfluidic sorter was fabricated using standard photolithography and soft lithography methods. Photomasks were designed using AutoCAD 2019. The coplanar sorter device, electrode, and dielectric layer fabrication followed standard photolithography procedures reported previously^32^. Briefly, chromium-coated glass slides (50 × 75 mm) with S1811 were exposed (5 s at 38–50 mW cm^−2^), developed in MF321 developer, etched with CR-4 chromium etchant, and stripped with an AZ-300T photoresist stripper. For the dielectric layer, the resulting patterned electrode substrate was placed under plasma oxygen (Harrick Plasma PDC-001, Ithaca, NY) for 1 min and 30 s, after which the samples were immediately spin-coated with an SU-8 5 layer (10 s, 500 rpm, 30 s 2250 rpm), soft-baked, and exposed to a sawtooth patterned mask. After baking, the substrates were developed, rinsed with isopropyl alcohol, and subjected to a hard baking cycle (180 °C, 10 min, gradual ramping). For the channel layer of the sorter device, a 4” Si wafer was treated under plasma oxygen for 1 min and 30 s. SU-8 2075 was spin-coated (500 rpm 10 s and 3250 rpm 30 s) to obtain a 70 µm layer, followed by a baking and exposure cycle according to the manufacturer’s datasheet. The second layer of SU-8 2075 was spin-coated on top of the undeveloped first layer (500 rpm for 10 s and 2250 rpm for 30 s) to obtain a 90 µm thick layer. After the pre-exposure bake, the second layer mask was feature-aligned and exposed (UV-KUB 2, Kloé, France), followed by baking and development according to the manufacturer’s datasheet. The resulting master mold was exposed to chlorotrimethylsilane vapor deposition in a desiccator for 45 min. PDMS (1:10 w/w ratio curing agent to prepolymer) was poured over the mold and left to cure in an oven (65 °C, 3 h). The PDMS layers were cut to size with an X-Acto knife. Inlets and outlets were made using 0.75 mm or 0.35 mm biopsy punchers (World Precision Instruments, FL, USA), fitting 1/32″ OD tubing or 360 µm OD tubing, respectively, after which the PDMS was carefully washed with IPA and ddH_2_O, air dried, and cleaned with tape to remove the dust before device assembly. The PDMS channel layer was treated with oxygen plasma for 30 s. Immediately after, the sorter was manually aligned with the dielectric-coated electrodes under a dissecting fluorescence microscope (Olympus IX73, 10X). The device channels were then treated with Novec 1720 fluorosilane polymer surfactant^32^. Two flat-cleaved multimode optical fibers were prepared for droplet excitation (100 µm core, 0.22 N.A.) and detection (200 µm core, 0.39 N.A.) (Thorlabs, NJ, US). The cladding was stripped off, and the fiber core was polished, cleaned, and carefully inserted into the respective optical fiber channel. The fibers were then fixed with Kapton tape. To retrieve droplets from the sorter, two 3 cm pieces of PEEK tubing (360 µm OD) were cut and treated with a similar Novec 1720 treatment. Outlet blockers were made by hot gluing one end of 1” PEEK 1/32″ OD tubing.

### Microfluidic sorting setup and operation

Gastight 500 μL glass syringes were prepared with fittings and tubing as reported previously^32^. The spore-containing syringe and the syringe for droplet reinjection were set up with 1/32″ OD 0.381 mm ID PEEK tubing. All other syringes had 1/32″ OD 0.127 mm ID tubing. Syringes were installed on a low-pressure neMESYS pump system (Cetoni, Korbussen, DE), and the spore-containing syringe was continuously stirred using a syringe stirrer (Nannostirus, V&P Scientific, San Diego, CA, USA). The sorting device with installed optical fibers was fixed in a 3D-printed holder and clamped in place with a pogo pin PCB providing contact with the electrode pads. The holder base plate fit in the scanning stage (XYZ Tango, Marzhauser, Wetzlar, DE) of an inverted epifluorescence microscope (Olympus IX78, Olympus, Montreal, Québec, CA). For a description of the Arduino-driven electrode control system, see the SI. Next, the SMA end of the excitation fiber was coupled to a 500 nm shortpass filter in an inline fiber optic filter mount (Thorlabs, NJ, US) connected to a high power (1 mW) 470 nm fiber-coupled LED light source. The SMA end of the emission fiber was coupled to a portable mini-spectrometer (FLAME-S UV‒VIS, Ocean Insight, NY, USA). The flow inside the microfluidic channel was observed under a ×4 or ×10 objective with bright-field illumination. The spectrometer, pressure-driven fluid flow, and electrode actuation were controlled using an in-house Python-based automation system and graphical user interface.

### Spectrometer data processing

Raw spectra were obtained by using the Seabreeze Python library and reading intensities [A.U.] in a threaded Python process. The SciPy signal processing library was used for spectrometer signal denoising and peak detection. Background subtraction was used to remove excitation signal noise and background light, and the absolute values of the resulting arbitrary fluorescence values were stored in a list as follows:

$$I = \left| {I_{n - 1} + (I_n-I_{{\mathrm{dark}}})} \right|,$$

where *I* is the raw intensity value from the spectrometer [RFU]. For signal denoising, a third-order Butterworth lowpass filter with 0.1 normalized cutoff frequency (*f*_*c*_) was applied to the intensity spectrum (user set). The processed arbitrary fluorescence values and gating area were plotted on a live plot. Peaks in the processed intensities list were detected based on height, peak base width, vertical distance to neighboring peaks, and peak prominence. Gated peaks were filtered to be within a certain wavelength and intensity range (user-set gate) above the user-set noise level. When the sorting process started, the CE was turned on, all peaks were detected, and the pulsing electrode (PE) was switched on when a gated peak was detected. All detected peaks were stored in a data file listing intensity and wavelength, which was converted to a.csv file to generate gating plots.

### Sorter characterization

Sorting efficiency was determined by a two-factor experiment with a binary response. Both the applied AC signal amplitude (10 kHz, sine wave, 50–550 mV_pp_, 7 levels) and the spacer oil speed (5–100 nL·s^−1^, 20 levels) were varied. Droplet generation was kept stable (water: 0.5 nL·s^−1^, oil: 1 nL·s^−1^). Droplets were sorted by actuating the pulsing electrode (PE) while keeping the negative channel electrode on (CE). Out of 10 sorting attempts, the number of successes was counted, and the sorting efficiency was calculated in terms of a percentage. To determine the effect of sorting polydisperse solutions, two conditions in the efficient sorting regime were chosen (350 mV_pp_, 60 nL·s^−1^, and 450 mV_pp_, 80 nL·s^−1^), and the droplet volume was varied. The droplet area was measured using Fiji (ImageJ) at an approximated height of 70 µm. To optimize the efficiency of autonomous sorting, the droplet travel time between the excitation point and sorting location was experimentally observed by varying the oil flow rate and recording a high-speed image series (30 msec/frame) (Hamamatsu Flash LT+ 4.0, Hamamatsu, JP). To optimize autonomous sorting, a mixed population of ddH_2_O and 50 µM or 100 µM analytical standard fluorescein droplets in 2% fluorosurfactant in HFE7500 oil (008-FluoroSurfactant, RAN Biotechnologies, Beverly, MA, USA) was generated using a dual T-junction droplet generator and transferred to the sorter using 360 µm OD PEEK capillary tubing. For each concentration of fluorescein, a gating histogram was made by recording 500 positive peaks above a set noise level within a wavelength range of 500–600 nm.

### Quantification of hyphal exiting from droplets

A *C. rosea* wild-type spore stock solution was filtered (10 µm), washed with DI water, centrifuged, and diluted in different media (CCMM, CCMM + 1% G, CCMM + 0.1% G, CCMM, and MM) to a final concentration of 1 × 10^5^ spores·mL^−1^. Droplets of single spores were generated by injecting the spore solution with media (30 nL·s^−1^) in a T-junction droplet generator (2% fluorosurfactant in HFE7500 oil at 60 nL·s^−1^). Next, droplets were collected in a PCR tube containing 50 µL of 2% fluorosurfactant in HFE7500 oil or glass capillaries and incubated at 27 °C for 4 days. Each day, the droplets in the PCR tube were pipetted onto a glass slide and photographed for droplet observation, and the capillaries were photographed to measure hyphae piercing through the droplets.

### Sorting and recovery of glycoside hydrolase-producing *C. rosea* strains

Before use, the colloidal chitin minimal medium (CCMM) was filtered through a 40 µm filter. For droplet incubation, mutant or wild-type spore stock solution was diluted in 2× CCMM to a final concentration of 0.35 × 10^6^ spores·mL^−1^ (*λ* = 0.35, Poisson distribution). Single-spore encapsulation was performed by mixing the spore solution (30 nL·s^-1^) with 200 µM fluorescein substrate (30 nL·s^−1^) using a microfluidic mixer T-junction droplet generator (1% fluorosurfactant in HFE7500 oil at 60 nL·s^−1^). Droplets were placed in a PCR tube containing 50 µL of 2% fluorosurfactant in HFE7500 oil and incubated in the dark at 27 °C for enzymatic production. After the appropriate incubation time, droplets were aspirated (0.2 µL·s^−1^) using a syringe pump and injected into the microfluidic sorter (0.01–0.02 µL·s^−1^). Autonomous sorting was performed at a sorting regime (oil flow rate and potential) that displayed efficient sorting. Droplets from the positive outlet were recovered in a capillary or PCR tube, plated on PDA (not more than 10–20 droplets per plate), and incubated at 27 °C. Mutant colonies were transferred to individual plates before neighboring hyphae touched (after ~3-4 days). Cultures were maintained for further well plate enzymatic assays. The glycoside hydrolase activity of the recovered mutants was confirmed with 4-MU substrates as described above.

### Modeling and data analysis

Data analysis was performed with Fiji, Python 3.9, GraphPad Prism v8.4.3, and R v3.6.2. The metadata from the high-speed camera were exported using Fiji by ImageJ and further analyzed with Python to determine the length of time required for a droplet to follow a specific path length. The droplet area was calculated using Fiji. The applied electrical signal was measured using an oscilloscope. Heat transfer, fluid, and electric field simulations were performed with COMSOL Multiphysics v5.4 (Supporting Information Note 2). All in-house codes were written in Python 3.9 and are published under GNU GPL v3.0 in our repository (https://bitbucket.org/shihmicrolab/fungalmicrofluidics/).

## Conclusion

In this study, a novel filamentous fungi high-throughput screening approach was developed using a solid-state fermentation droplet incubation method and an electrostatic low-voltage droplet microfluidic sorter. The system was established using *C. rosea* and successfully screened for high-performing cell-wall-degrading enzymes. Compared with traditional screening methods available for the high-throughput screening of filamentous fungi, this method greatly increased the screening speed and reduced the labor and tedium associated with testing proteins secreted from fungi. To our knowledge, this is the first work on filamentous fungi microfluidic sorting that shows advancements in long-term droplet culture based on solid-state fermentation and a sorting method specifically designed to handle fungal droplet libraries. Compared to the currently available filamentous fungi screening techniques, the 7 Hz throughput in this study is faster than the macroscale high-throughput well plate-based methods and comparable to the previously reported microfluidic methods for fungal screening. Given our longer incubation times (4 days), we resolved the enzymatic activity in *C. rosea*, which was previously difficult to demonstrate with single-spore libraries. We believe our system is the first step to further explore solid-state fermentation methods in nano- or picoliter droplets for continued investigation of the effect of long-term incubation on screening methods. Beyond electrostatic or DEP-based sorting, the screening strategy we present here could be applied to other filamentous fungal strains and enzymes using other fluorescein-based substrates and can thus be used for many industrial biotechnology applications.

## Supplementary information

Supplemental Information

## References

[1] Wösten HAB (2019). Filamentous fungi for the production of enzymes, chemicals and materials. Curr. Opin. Biotechnol..

[2] Copetti MV (2019). Fungi as industrial producers of food ingredients. Curr. Opin. Food Sci..

[3] Chan LG, Cohen JL, de Moura Bell JMLN (2018). Conversion of agricultural streams and food-processing by-products to value-added compounds using filamentous fungi. Annu. Rev. Food Sci. Technol..

[4] Schroers H-J, Samuels GJ, Seifert KA, Gams W (1999). Classification of the Mycoparasite Gliocladium roseum in Clonostachys as C. rosea, its relationship to Bionectria ochroleuca, and notes on other gliocladium-like fungi. Mycologia.

[5] Jensen DF (2007). Development of a biocontrol agent for plant disease control with special emphasis on the near commercial fungal antagonist Clonostachys rosea strain ‘IK726’. Australas. Plant Pathol..

[6] Sun Z-B (2020). Biology and applications of Clonostachys rosea. J. Appl. Microbiol..

[7] Karlsson M (2015). Insights on the evolution of mycoparasitism from the genome of Clonostachys rosea. Genome Biol. Evol..

[8] Tzelepis G, Dubey M, Jensen DF, Karlsson M (2015). Identifying glycoside hydrolase family 18 genes in the mycoparasitic fungal species Clonostachys rosea. Microbiology.

[9] Sun, Z.-B., Sun, M.-H. & Li, S.-D. Draft genome sequence of Mycoparasite Clonostachys rosea strain 67-1. *Genome Announc.* **3**, e00546–15 (2015). 10.1128/genomeA.00546-15 PMC4447911 26021926

[10] Nevalainen H, Peterson R, Curach N (2018). Overview of gene expression using filamentous fungi. Curr. Protoc. Protein Sci..

[11] Kluge J, Terfehr D, Kück U (2018). Inducible promoters and functional genomic approaches for the genetic engineering of filamentous fungi. Appl. Microbiol. Biotechnol..

[12] Bleichrodt R-J, Read ND (2019). Flow cytometry and FACS applied to filamentous fungi. Fungal Biol. Rev..

[13] Mathis H, Margeot A, Bouix M (2020). Optimization of flow cytometry parameters for high-throughput screening of spores of the filamentous fungus Trichoderma reesei. J. Biotechnol..

[14] Gielen F (2016). Ultrahigh-throughput–directed enzyme evolution by absorbance-activated droplet sorting (AADS). PNAS.

[15] Kim HS (2017). High-throughput droplet microfluidics screening platform for selecting fast-growing and high lipid-producing microalgae from a mutant library. Plant Direct.

[16] Vallejo D, Nikoomanzar A, Paegel BM, Chaput JC (2019). Fluorescence-activated droplet sorting for single-cell directed evolution. ACS Synth. Biol..

[17] Li M (2018). A gelatin microdroplet platform for high-throughput sorting of hyperproducing single-cell-derived microalgal clones. Small.

[18] Baret J-C (2009). Fluorescence-activated droplet sorting (FADS): efficient microfluidic cell sorting based on enzymatic activity. Lab Chip.

[19] Hébert M, Courtney M, Ren CL (2019). Semi-automated on-demand control of individual droplets with a sample application to a drug screening assay. Lab Chip.

[20] Sjostrom SL (2014). High-throughput screening for industrial enzyme production hosts by droplet microfluidics. Lab Chip.

[21] Shembekar N, Hu H, Eustace D, Merten CA (2018). Single-cell droplet microfluidic screening for antibodies specifically binding to target cells. Cell Rep..

[22] Mahler L (2018). Detection of antibiotics synthetized in microfluidic picolitre-droplets by various actinobacteria. Sci. Rep..

[23] Zang E (2013). Real-time image processing for label-free enrichment of Actinobacteria cultivated in picolitre droplets. Lab Chip.

[24] Tu R (2021). Droplet-based microfluidic platform for high-throughput screening of Streptomyces. Commun. Biol..

[25] He R, Ding R, Heyman JA, Zhang D, Tu R (2019). Ultra-high-throughput picoliter-droplet microfluidics screening of the industrial cellulase-producing filamentous fungus Trichoderma reesei. J. Ind. Microbiol. Biotechnol..

[26] Beneyton T (2016). High-throughput screening of filamentous fungi using nanoliter-range droplet-based microfluidics. Sci. Rep..

[27] Millet LJ (2019). Increasing access to microfluidics for studying fungi and other branched biological structures. Fungal Biol. Biotechnol..

[28] Xi H-D (2017). Active droplet sorting in microfluidics: a review. Lab Chip.

[29] Pit AM (2015). High-throughput sorting of drops in microfluidic chips using electric capacitance. Biomicrofluidics.

[30] Ruiter Rde (2014). Electrostatic potential wells for on-demand drop manipulation in microchannels. Lab Chip.

[31] Ahmadi F, Samlali K, Vo PQN, Shih SCC (2019). An integrated droplet-digital microfluidic system for on-demand droplet creation, mixing, incubation, and sorting. Lab Chip.

[32] Samlali K, Ahmadi F, Quach ABV, Soffer G, Shih SCC (2020). One cell, one drop, one click: hybrid microfluidics for mammalian single cell isolation. Small.

[33] Demissie ZA (2020). Transcriptomic and exometabolomic profiling reveals antagonistic and defensive modes of Clonostachys rosea action against Fusarium graminearum. Mol. Plant Microbe Interact..

[34] Cole RH, de Lange N, Gartner ZJ, Abate AR (2015). Compact and modular multicolour fluorescence detector for droplet microfluidics. Lab Chip.

[35] Holstein JM, Gylstorff C, Hollfelder F (2021). Cell-free directed evolution of a protease in microdroplets at ultrahigh throughput. ACS Synth. Biol..

[36] Saito K (2021). Microdroplet-based system for culturing of environmental microorganisms using FNAP-sort. Sci. Rep..

[37] Isozaki A (2020). Sequentially addressable dielectrophoretic array for high-throughput sorting of large-volume biological compartments. Sci. Adv..

[38] Chatterton S, Punja ZK (2009). Chitinase and beta-1,3-glucanase enzyme production by the mycoparasite Clonostachys rosea f. catenulata against fungal plant pathogens. Can. J. Microbiol..

[39] Aita BC (2019). Production of cell-wall degrading enzymes by solid-state fermentation using agroindustrial residues as substrates. J. Environ. Chem. Eng..

[40] Utech S (2015). Microfluidic generation of monodisperse, structurally homogeneous alginate microgels for cell encapsulation and 3D cell culture. Adv. Healthc. Mater..

[41] Napiorkowska M, Pestalozzi L, Panke S, Held M, Schmitt S (2021). High-throughput optimization of recombinant protein production in microfluidic gel beads. Small.

[42] Eun Y-J, Utada AS, Copeland MF, Takeuchi S, Weibel DB (2011). Encapsulating bacteria in agarose microparticles using microfluidics for high-throughput cell analysis and isolation. ACS Chem. Biol..

[43] Lin Y-S, Yang C-H, Lu K, Huang K-S, Zheng Y-Z (2011). Synthesis of agar microparticles using temperature-controlled microfluidic devices for Cordyceps militaris cultivation. Electrophoresis.

[44] Amselem G, Guermonprez C, Drogue B, Michelin S, Baroud CN (2016). Universal microfluidic platform for bioassays in anchored droplets. Lab Chip.

[45] Chandler, D. in *Microbial Control of Insect and Mite Pests* (ed. Lacey, L. A.) Ch. 5, 69–89 (Academic Press, 2017).

[46] Merino N (2018). Fungal biotransformation of 6:2 fluorotelomer alcohol. Remediation.

[47] Prenafeta-Boldú FX, Luykx DMAM, Vervoort J, de Bont JAM (2001). Fungal metabolism of toluene: monitoring of fluorinated analogs by ^19^ F nuclear magnetic resonance spectroscopy. Appl. Environ. Microbiol.

[48] Kiel M, Engesser K-H (2015). The biodegradation vs. biotransformation of fluorosubstituted aromatics. Appl. Microbiol. Biotechnol..

[49] Innocenti G, Roberti R, Montanari M, Zakrisson E (2003). Efficacy of microorganisms antagonistic to Rhizoctonia cerealis and their cell wall degrading enzymatic activities. Mycol. Res..

[50] Baret J-C (2009). Fluorescence-activated droplet sorting (FADS): efficient microfluidic cell sorting based on enzymatic activity. Lab Chip.

[51] Kintses B (2012). Picoliter cell lysate assays in microfluidic droplet compartments for directed enzyme evolution. Chem. Biol..

[52] Fevre M (1979). Intracellular and cell wall associated (1 → 3) β glucanases of Saprolegnia. Mycopathologia.

[53] Tingle MA, Halvorson HO (1971). A comparison of β-glucanase and β-glucosidase in Saccharomyces lactis. Biochim. Biophys. Acta Enzymol..

[54] Gastebois A (2013). SUN proteins belong to a novel family of β-(1,3)-glucan-modifying enzymes involved in fungal morphogenesis. J. Biol. Chem..

[55] Lafond M, Navarro D, Haon M, Couturier M, Berrin J-G (2012). Characterization of a broad-specificity β-glucanase acting on β-(1,3)-, β-(1,4)-, and β-(1,6)-glucans that defines a new glycoside hydrolase family. Appl. Environ. Microbiol..

[56] Clark IC, Thakur R, Abate AR (2018). Concentric electrodes improve microfluidic droplet sorting. Lab Chip.

[57] Sciambi A, Abate AR (2014). Accurate microfluidic sorting of droplets at 30 kHz. Lab Chip.

[58] Karlsson, M., Atanasova, L., Jensen, D. F. & Zeilinger, S. Necrotrophic mycoparasites and their genomes. *Microbiol. Spectr*. **5**, 5.2.08 (2017). 10.1128/microbiolspec.funk-0016-2016 PMC11687461 28281442

[59] Mania D, Hilpert K, Ruden S, Fischer R, Takeshita N (2010). Screening for antifungal peptides and their modes of action in Aspergillus nidulans. Appl. Environ. Microbiol..

[60] Wu Y-J, Cheng C-Y, Li Y-K (2009). Cloning and expression of Chitinase A from Serratia Marcescens for large-scale preparation of N,N-diacetyl chitobiose. J. Chin. Chem. Soc..

[61] Subramanian K (2020). Bioconversion of chitin and concomitant production of chitinase and N-acetylglucosamine by novel Achromobacter xylosoxidans isolated from shrimp waste disposal area. Sci. Rep..

