# Supplementary material for: Droplet digital microfluidic system for screening filamentous fungi based on enzymatic activity
Source: Microsyst Nanoeng. 2022 Nov 21;8:123. doi: 10.1038/s41378-022-00456-1 (PMC9681769; doi:10.1038/s41378-022-00456-1)
Supplement: Supplementary file 1 — Supplemental Information [file 41378_2022_456_MOESM1_ESM.docx]

**Supporting Information**

**Droplet digital microfluidic system for screening filamentous fungi based on enzymatic activity**

Kenza Samlali,^1, 2^ Chiara Leal Alves,^1, 2^ Mara Jezernik,^3^ Steve C.C. Shih ^1,2,4^*

^1^ Department of Electrical and Computer Engineering, Concordia University, Montréal, Québec, Canada

^2^ Centre for Applied Synthetic Biology, Concordia University, Montréal, Québec, Canada

^3^ Department of Chemical Engineering and Applied Chemistry, University of Toronto, Toronto, Ontario, Canada

^4^ Department of Biology, Concordia University, Montréal, Québec, Canada

*Corresponding author

Tel: +1-(514) 848-2424 x7579

Email: steve.shih@concordia.ca

**Supporting Information and Notes**

**Note 1: Device and automation system**

In **Figure 1**, the channel-part of the microfluidic device was fabricated using PDMS with a height of 70 µm. For the electrode layer, the electrode gap is 15 µm, and a dielectric (SU-8 5) of 7 µm (smaller than half the size of the gap) was deposited on top. All other dimensions and architecture can be found in **Supplementary Figure 1**.

To apply potentials for sorting droplets, an AC potential was generated using a function generator (33201A Agilent, Allied Electronics, Ottawa, ON) and an amplifier (PZD-700A, Trek Inc., Lockport, NY) and sent to a custom board with Arduino UNO controlled optocoupler switches. For a detailed hardware overview, we refer to our previous work. Pump (Nemesys CETONI) and electrode operation were driven using python based software as described in our previous work.^1^ In addition, background subtraction, gating and sorting were all performed through our Python based open-source software with GUI (GNU GPLv3, <https://bitbucket.org/shihmicrolab/fungalmicrofluidics.git>) (**Supplementary Figure 13**). Seabreeze was used as a Python library for communication with the spectrometer, allowing for compatibility of this software with several other affordable spectrometers (<https://python-seabreeze.readthedocs.io>). The graphical user interface consisted out of two main windows: one window contained the control interface, and another window contained a plot viewer with a raw spectrum and a processed spectrum (denoised and indicating the sorting gate) (**Supplementary** **Figure 14**).

**Note 2: Spectrum detection and signal processing**

To eliminate the background signal, we initially placed a band-pass filter in-line between the spectrometer and the outgoing 200 µm emission optical fiber. For peak detection, a program was created for detecting peaks that have a peak height above a user-defined threshold intensity. Although background noise was removed, the bandpass filter reduced the intensity of the peaks and useful information outside the band-pass filter range was lost. For example, air droplets reflected excitation light and showed peaks with a large base (> 200 nm) and high intensity often overlapping with the sample bandpass range. Air droplets were accidentally misidentified as droplets within the sorting gate. We finally opted to not use an emission bandpass filter and use a background subtraction method and peak detection algorithm instead. A background frame of experimental light conditions (excitation light, background light, noise, with oil sample) were recorded and subtracted from future frames (**Supplementary** **Figure 3**). The peak detection algorithm detects peaks (local maxima) in a subset (gate) of the spectrum. By setting the peak height range, peak base width, a wavelength and maximum peak height gate, and distance to neighboring peak, reliable detection of emission spectra could be performed. The resulting spectrum provided useful information on the sample type. As such, this peak detection method offers several advantages over specific wavelength based raw intensity detection performed with PMT’s.

**Table 1. COMSOL electric field model parameters**

| **Property** | **Value** | **Unit** |
| --- | --- | --- |
| PDMS relative permittivity | 2.75 |  |
| PDMS electrical conductivity | 4x10^-13^ | S/m |
| HFE 7500 oil relative permittivity | 5.8 |  |
| HFE 7500 electrical conductivity | 3x10^-8^ | S/m |
| Dielectric thickness SU-8 5 | 7.0 | µm |
| SU8-5 relative Permittivity | 4 |  |
| SU8 electrical conductivity | 2.8x10^-14^ | S/m |

**Table 2. Comparison of system needs between typical FADS sorting setup and our system.**

|  | **Equipment** | |
| --- | --- | --- |
| **Purpose** | **Typical FADS sorting setup** | **Samlali et al. sorting setup** |
| Excitation | Laser, microscope, objectives for focusing | High power LED, optical fiber, in-line short pass filter |
| Detection | PMT | Optical fiber, mini-spectrometer |
| High-voltage electronics | Amplifier, function generator, high-volage and frequency switch board | None – only require low-voltage electronics (< 36 V) |
| Sorting efficiency | >90% | ~86% |
| Estimated costs | >$10k | $5k (most expensive is the spectrometer) |
| Droplet size | Picoliter range | Picoliter and nanoliter range |
| Throughput | Up to 20kHz | 7-20Hz |
| Droplet morphology | Uniform | Polydisperse |

**Table 3. Comparison of other fungi screening devices and our system.**

|  | **Sorting parameters** | | |
| --- | --- | --- | --- |
| **Purpose** | **He, et al. 2019** | **Beneyton, et al. 2016** | **Samlali et al.** |
| Screening marker | Cellulases (*T. reesei*) | α-Amylase (*A. niger*) | Chitinase and N- Acetylgalactosaminidase (*Cl. Rosea*) |
| Throughput | ~ 3 Hz (10,000 droplets/h) | 10 Hz | ~ 7 Hz (25,000 droplets/h) |
| Sorting mechanism | DEP | DEP | Electrostatic-based sorting |
| Incubation time | 12 hpi | 24hpi | 96hpi |
| Droplet size | ~ 1 nL (100 µm diameter) | 18 nL | Polydisperse (~ 0.5 – 1.5 nL) |
| Fold improvement | ~1.5 fold | ~2.3 fold | ~4 fold |

**Table 4.** Slope of tip exiting overtime expressed in % hpi^-1^.

| **Media composition** | **Slope** | **Std Error** |
| --- | --- | --- |
| 1% G + MM | 1.04 | 0.1254 |
| 1% CC + 1% G + MM | 0.8952 | 0.1697 |
| MM | 0.1079 | 0.06106 |
| 1% CC + 0.1% G + MM | 0.05128 | 0.01451 |

**Table 5.** One-way ANOVA (Dunnett's multiple comparisons test) of Rate of tip exiting [% hpi^-1^]. P values: 0.1234 (ns), 0.0332 (*), 0.0021 (**), 0.0002 (***), <0.0001 (****).

| **Comparison** | **Significance** | **P Adj** |
| --- | --- | --- |
| GMM vs. CCMM + 1% G | **** | <0.0001 |
| GMM vs. MM | **** | <0.0001 |
| GMM vs. CCMM + 0.1% G | **** | <0.0001 |
| CCMM + 1% G vs. MM | **** | <0.0001 |
| CCMM + 1% G vs. CCMM + 0.1% G | **** | <0.0001 |
| MM vs. CCMM + 0.1% G | ns | 0.2267 |

**Table 6. Confusion matrix evaluation measures**

| **Measure** |  | **Value** |
| --- | --- | --- |
| Error rate | ERR | 0.03703704 |
| Accuracy | ACC | 0.96296296 |
| Sensitivity | SN | 0.77142857 |
| True positive rate | TPR |  |
| Recall | REC |  |
| Specificity | SP | 0.99519231 |
| True negative rate | TNR |  |
| Precision | PREC | 0.96428571 |
| Positive predictive value | PPV |  |
| False positive rate | FPR | 0.00480769 |
| Matthews Correlation coefficient | MCC | 127.202795 |
| F-score | F | 5 |
|  | Beta | 0.5 |
|  |  |  |

**Table 7**. One-way ANOVA test of enzymatic activity of **N-acetyl galactosaminidase** overtime produced by recovered strains grown in **GMM** compared to the wild type. P values: 0.1234 (ns), 0.0332 (*), 0.0021 (**), 0.0002 (***), <0.0001 (****).

| **Strain** | **0 days** | | | | **2 days** | | | | **4 days** | | | |
| --- | --- | --- | --- | --- | --- | --- | --- | --- | --- | --- | --- | --- |
|  | **Mean** | **StDev** | **Sig** | **P Adj** | **Mean** | **StDev** | **Sig** | **P Adj** | **Mean** | **StDev** | **Sig** | **P Adj** |
| MC1 | 0.009739 | 0.005423 | ns | >0.9999 | 0.9105 | 1.02 | ns | 0.9993 | 1.409 | 0.9652 | ns | 0.9989 |
| MC2 | 0 | 0 | ns | >0.9999 | 0.1546 | 0.0369 | ns | >0.9999 | 1.696 | 0.4029 | ns | 0.9945 |
| MC3 | 0.007939 | 0.008399 | ns | >0.9999 | 0.01054 | 0.01023 | ns | >0.9999 | 0.3044 | 0.02672 | ns | >0.9999 |
| MC4 | 0 | 0 | ns | >0.9999 | 0.0474 | 0.02469 | ns | >0.9999 | 0.6628 | 0.4233 | ns | 0.9996 |
| MC5 | 0.06032 | 0.00555 | ns | 0.9998 | 0.5944 | 0.06037 | ns | 0.9996 | 0.9669 | 0.1497 | ns | 0.9994 |
| MC6 | 0.7287 | 0.3971 | ns | 0.1251 | 1.304 | 0.72 | ns | 0.9955 | 2.535 | 0.9272 | ns | 0.8793 |
| MC7 | 0.8145 | 0.5184 | ns | 0.0588 | 3.316 | 1.623 | ns | 0.3833 | 5.675 | 3.471 | * | 0.0329 |
| MG1 | 0.4983 | 0.2795 | ns | 0.5827 | 4.232 | 2.633 | ns | 0.1228 | 2.984 | 2.049 | ns | 0.7045 |
| MG2 | 0.6031 | 0.1601 | ns | 0.3186 | 4.559 | 0.6821 | ns | 0.0756 | 5.682 | 1.022 | * | 0.0326 |
| MG3 | 0.07201 | 0.07644 | ns | 0.9997 | 1.682 | 0.3679 | ns | 0.9866 | 1.186 | 0.9199 | ns | 0.9991 |
| MG4 | 0.2613 | 0.01513 | ns | 0.9943 | 11.83 | 2.146 | **** | <0.0001 | 13.03 | 2.606 | **** | <0.0001 |
| MG5 | 2.141 | 1.657 | **** | <0.0001 | 9.828 | 9.46 | **** | <0.0001 | 11.85 | 9.73 | **** | <0.0001 |
| MG6 | 0.3579 | 0.03329 | ns | 0.9287 | 5.368 | 1.159 | * | 0.0196 | 8.599 | 2.644 | *** | 0.0002 |
| MG7 | 0.001594 | 0.002401 | ns | >0.9999 | 0.03187 | 0.01305 | ns | >0.9999 | 0.9459 | 0.5497 | ns | 0.9994 |
| MG8 | 0 | 0 | ns | >0.9999 | 0.06389 | 0.04808 | ns | >0.9999 | 1.77 | 0.8474 | ns | 0.9941 |
| MG9 | 0.001771 | 0.00262 | ns | >0.9999 | 0.1668 | 0.0669 | ns | >0.9999 | 0.6672 | 0.4389 | ns | 0.9996 |
| MG10 | 0.000443 | 0.000886 | ns | >0.9999 | 0.2941 | 0.243 | ns | 0.9998 | 0.82 | 0.1891 | ns | 0.9995 |
| MG11 | 0 | 0 | ns | >0.9999 | 0 | 0 | ns | >0.9999 | 0.1232 | 0.01103 | ns | >0.9999 |
| MG12 | 0.004457 | 0.003382 | ns | >0.9999 | 0.4014 | 0.3191 | ns | 0.9997 | 1.792 | 1.455 | ns | 0.9897 |
| MG13 | 0 | 0 | ns | >0.9999 | 0.02444 | 0.02913 | ns | >0.9999 | 0.3511 | 0.3706 | ns | 0.9999 |
| **wt** | **0.01703** | **0.01807** |  |  | **0.0623** | **0.0457** |  |  | **0.1675** | **0.01074** |  |  |

**Table 8.** One-way ANOVA test of enzymatic activity of **chitinase** overtime produced by recovered strains grown in **GMM** compared to the wild type. P values: 0.1234 (ns), 0.0332 (*), 0.0021 (**), 0.0002 (***), <0.0001 (****).

| **Strain** | **0 days** | | | | **2 days** | | | | **4 days** | | | |
| --- | --- | --- | --- | --- | --- | --- | --- | --- | --- | --- | --- | --- |
|  | **Mean** | **StDev** | **Sig** | **P Adj** | **Mean** | **StDev** | **Sig** | **P Adj** | **Mean** | **StDev** | **Sig** | **P Adj** |
| MC1 | 0.001107 | 0.000995 | ns | >0.9999 | 1.874 | 2.003 | ns | 0.9386 | 2.284 | 1.533 | ns | 0.728 |
| MC2 | 0 | 0 | ns | >0.9999 | 0.5933 | 0.08095 | ns | 0.9997 | 3.055 | 0.693 | ns | 0.2972 |
| MC3 | 0.006257 | 0.000849 | ns | >0.9999 | 0.06747 | 0.004672 | ns | 0.9998 | 0.5119 | 0.03766 | ns | 0.9998 |
| MC4 | 0.003586 | 0.004125 | ns | >0.9999 | 0.2745 | 0.0317 | ns | >0.9999 | 1.21 | 0.8243 | ns | 0.9989 |
| MC5 | 0.1115 | 0.009706 | ns | 0.9996 | 1.824 | 0.07066 | ns | 0.9519 | 1.669 | 0.3144 | ns | 0.9766 |
| MC6 | 1.21 | 0.6645 | * | 0.0157 | 3.148 | 1.685 | ns | 0.2903 | 3.334 | 2.503 | ns | 0.1933 |
| MC7 | 1.18 | 0.2818 | * | 0.0201 | 6.971 | 2.595 | **** | <0.0001 | 7.411 | 3.481 | **** | <0.0001 |
| MG1 | 0.8632 | 0.4955 | ns | 0.1888 | 7.861 | 3.582 | **** | <0.0001 | 6.964 | 2.391 | **** | <0.0001 |
| MG2 | 1.059 | 0.1688 | ns | 0.0513 | 9.534 | 0.8587 | **** | <0.0001 | 8.173 | 1.057 | **** | <0.0001 |
| MG3 | 0.1335 | 0.1347 | ns | 0.9996 | 3.546 | 0.6804 | ns | 0.1578 | 1.656 | 0.9794 | ns | 0.9785 |
| MG4 | 0.3981 | 0.03502 | ns | 0.9762 | 13.17 | 0.6786 | **** | <0.0001 | 12.05 | 0.6693 | **** | <0.0001 |
| MG5 | 2.957 | 2.118 | **** | <0.0001 | 9.253 | 6.149 | **** | <0.0001 | 9.671 | 5.08 | **** | <0.0001 |
| MG6 | 0.6673 | 0.06918 | ns | 0.5098 | 10.31 | 1.35 | **** | <0.0001 | 10.71 | 1.46 | **** | <0.0001 |
| MG7 | 0.000384 | 0.000483 | ns | >0.9999 | 0.195 | 0.04369 | ns | >0.9999 | 1.601 | 0.937 | ns | 0.9855 |
| MG8 | 0 | 0 | ns | >0.9999 | 0.3435 | 0.01737 | ns | >0.9999 | 3.187 | 1.126 | ns | 0.2439 |
| MG9 | 0.000561 | 0.000709 | ns | >0.9999 | 0.5857 | 0.1131 | ns | 0.9997 | 1.101 | 0.7507 | ns | 0.999 |
| MG10 | 0 | 0 | ns | >0.9999 | 0.8523 | 0.6241 | ns | 0.9994 | 1.399 | 0.3528 | ns | 0.9944 |
| MG11 | 0 | 0 | ns | >0.9999 | 0.004604 | 0.003726 | ns | 0.9997 | 0.1731 | 0.00843 | ns | 0.9999 |
| MG12 | 0.01328 | 0.01009 | ns | >0.9999 | 1.076 | 0.8229 | ns | 0.9991 | 2.911 | 2.352 | ns | 0.3638 |
| MG13 | 0 | 0 | ns | >0.9999 | 0.1554 | 0.1237 | ns | 0.9999 | 0.2531 | 0.03859 | ns | >0.9999 |
| **wt** | **0.01434** | **0.01258** |  |  | **0.2729** | **0.04273** |  |  | **0.2885** | **0.03039** |  |  |

**Table 9.** One-way ANOVA test of enzymatic activity of **glucanases** overtime produced by recovered strains grown in **GMM** compared to the wild type. P values: 0.1234 (ns), 0.0332 (*), 0.0021 (**), 0.0002 (***), <0.0001 (****).

| **Strain** | **0 days** | | | | **2 days** | | | | **4 days** | | | |
| --- | --- | --- | --- | --- | --- | --- | --- | --- | --- | --- | --- | --- |
|  | **Mean** | **StDev** | **Sig** | **P Adj** | **Mean** | **StDev** | **Sig** | **P Adj** | **Mean** | **StDev** | **Sig** | **P Adj** |
| MC1 | 0.03112 | 0.02674 | ns | 0.9997 | 2.304 | 2.132 | ns | 0.9989 | 4.087 | 3.199 | ns | 0.9954 |
| MC2 | 0.1132 | 0.07955 | ns | 0.9999 | 0.3242 | 0.2584 | ns | >0.9999 | 1.236 | 0.1666 | ns | 0.9998 |
| MC3 | 2.043 | 3.701 | * | 0.0229 | 0.2705 | 0.13 | ns | 0.9999 | 0.7327 | 0.1643 | ns | 0.9996 |
| MC4 | 0.1771 | 0.1875 | ns | >0.9999 | 0.4018 | 0.1115 | ns | >0.9999 | 1.141 | 0.2613 | ns | 0.9997 |
| MC5 | 0.3369 | 0.2997 | ns | 0.9996 | 2.731 | 0.6181 | ns | 0.9945 | 3.159 | 1.061 | ns | 0.9993 |
| MC6 | 0.8148 | 0.08427 | ns | 0.9671 | 1.524 | 0.08912 | ns | 0.9994 | 2.29 | 0.9146 | ns | 0.9997 |
| MC7 | 0.5397 | 0.1239 | ns | 0.999 | 2.571 | 0.887 | ns | 0.9952 | 3.006 | 1.212 | ns | 0.9994 |
| MG1 | 0.5112 | 0.1786 | ns | 0.9991 | 5.903 | 1.07 | ns | 0.3282 | 5.819 | 0.7917 | ns | 0.8704 |
| MG2 | 0.5385 | 0.1603 | ns | 0.999 | 4.788 | 0.3046 | ns | 0.6406 | 5.133 | 0.4703 | ns | 0.9658 |
| MG3 | 0.4105 | 0.2247 | ns | 0.9994 | 4.663 | 1.984 | ns | 0.6794 | 1.722 | 1.054 | ns | >0.9999 |
| MG4 | 0.6005 | 0.1187 | ns | 0.9958 | 36.91 | 13.21 | **** | <0.0001 | 31.16 | 9.248 | **** | <0.0001 |
| MG5 | 1.233 | 0.7898 | ns | 0.5078 | 5.502 | 4.713 | ns | 0.4292 | 16.59 | 16.85 | **** | <0.0001 |
| MG6 | 0.2326 | 0.1096 | ns | 0.9999 | 4.507 | 1.518 | ns | 0.7268 | 6.112 | 1.887 | ns | 0.8087 |
| MG7 | 0.02222 | 0.0437 | ns | 0.9997 | 0.01904 | 0.03807 | ns | 0.9997 | 0.7039 | 0.2742 | ns | 0.9996 |
| MG8 | 0.003497 | 0.004859 | ns | 0.9996 | 0.03338 | 0.02476 | ns | 0.9997 | 1.242 | 0.3318 | ns | 0.9998 |
| MG9 | 0.04548 | 0.06954 | ns | 0.9997 | 4.58 | 7.527 | ns | 0.7048 | 1.606 | 0.123 | ns | >0.9999 |
| MG10 | 0.03019 | 0.05386 | ns | 0.9997 | 0.4212 | 0.3341 | ns | >0.9999 | 0.9178 | 0.01745 | ns | 0.9997 |
| MG11 | 0 | 0 | ns | 0.9996 | 0 | 0 | ns | 0.9997 | 0.1212 | 0.01414 | ns | 0.9993 |
| MG12 | 0.2439 | 0.1567 | ns | 0.9998 | 2.53 | 1.132 | ns | 0.9954 | 2.656 | 0.8471 | ns | 0.9996 |
| MG13 | 0.02255 | 0.03903 | ns | 0.9997 | 0 | 0 | ns | 0.9997 | 0.2136 | 0.05979 | ns | 0.9993 |
| **wt** | **0.1647** | **0.1603** |  |  | **0.5244** | **0.3566** |  |  | **1.723** | **0.6465** |  |  |

**Table 10.** One-way ANOVA test of enzymatic activity of **N-acetyl Galactosaminidase** overtime produced by recovered strains grown in **CCMM** compared to the wild type. P values: 0.1234 (ns), 0.0332 (*), 0.0021 (**), 0.0002 (***), <0.0001 (****).

| **Strain** | **0 days** | | | | **2 days** | | | | **4 days** | | | |
| --- | --- | --- | --- | --- | --- | --- | --- | --- | --- | --- | --- | --- |
|  | **Mean** | **StDev** | **Sig** | **P Adj** | **Mean** | **StDev** | **Sig** | **P Adj** | **Mean** | **StDev** | **Sig** | **P Adj** |
| MC1 | 0.01889 | 0.004315 | ns | >0.9999 | 0.7135 | 0.7974 | ns | 0.9997 | 1.225 | 1.165 | ns | 0.9997 |
| MC2 | 0.01363 | 0.00113 | ns | >0.9999 | 0.04362 | 0.01206 | ns | >0.9999 | 0.0484 | 0.02136 | ns | 0.9999 |
| MC3 | 0.01039 | 0.004276 | ns | >0.9999 | 0.02149 | 0.01039 | ns | >0.9999 | 0.03317 | 0.01701 | ns | 0.9999 |
| MC4 | 0.01665 | 0.005047 | ns | >0.9999 | 0.033 | 0.01165 | ns | >0.9999 | 0.1399 | 0.05151 | ns | >0.9999 |
| MC5 | 0.02296 | 0.003075 | ns | >0.9999 | 0.06003 | 0.04204 | ns | >0.9999 | 0.2091 | 0.03666 | ns | >0.9999 |
| MC6 | 0.09149 | 0.02676 | ns | >0.9999 | 3.321 | 3.367 | ns | 0.9864 | 4.448 | 4.07 | ns | 0.9399 |
| MC7 | 0.3086 | 0.2087 | ns | 0.394 | 0.03837 | 0.02085 | ns | >0.9999 | 0.1015 | 0.0513 | ns | >0.9999 |
| MG1 | 0.44 | 0.2716 | * | 0.0273 | 8.133 | 6.784 | ns | 0.1381 | 17.28 | 9.162 | **** | <0.0001 |
| MG2 | 0.6249 | 0.08995 | *** | 0.0002 | 4.184 | 1.696 | ns | 0.9073 | 5.098 | 1.796 | ns | 0.8419 |
| MG3 | 0.3836 | 0.287 | ns | 0.0985 | 13.91 | 15.84 | *** | 0.0006 | 14.44 | 16.22 | ** | 0.0013 |
| MG4 | 1.718 | 0.4391 | **** | <0.0001 | 5.994 | 0.4241 | ns | 0.4966 | 6.49 | 0.5441 | ns | 0.5339 |
| MG5 | 1.035 | 0.3843 | **** | <0.0001 | 4.374 | 2.344 | ns | 0.8754 | 3.338 | 0.8101 | ns | 0.9943 |
| MG6 | 0.4053 | 0.09588 | ns | 0.0613 | 4.041 | 2.319 | ns | 0.9279 | 4.987 | 2.502 | ns | 0.8621 |
| MG7 | 0.01428 | 0.009693 | ns | >0.9999 | 0.3783 | 0.4269 | ns | >0.9999 | 0.4642 | 0.3514 | ns | >0.9999 |
| MG8 | 0.01145 | 0.002116 | ns | >0.9999 | 0.01936 | 0.008799 | ns | >0.9999 | 0.08812 | 0.07607 | ns | >0.9999 |
| MG9 | 0.02473 | 0.000621 | ns | >0.9999 | 0.1629 | 0.1496 | ns | >0.9999 | 0.2249 | 0.1445 | ns | >0.9999 |
| MG10 | 0.02733 | 0.001473 | ns | >0.9999 | 0.08889 | 0.03623 | ns | >0.9999 | 0.201 | 0.04774 | ns | >0.9999 |
| MG11 | 0.0118 | 0.01318 | ns | >0.9999 | 0.004029 | 0.003115 | ns | >0.9999 | 0.1022 | 0.1092 | ns | >0.9999 |
| MG12 | 0.3524 | 0.3943 | ns | 0.1851 | 0.1647 | 0.05105 | ns | >0.9999 | 0.2878 | 0.1397 | ns | >0.9999 |
| MG13 | 0.03583 | 0.00311 | ns | >0.9999 | 0.01895 | 0.01087 | ns | >0.9999 | 0.2533 | 0.1052 | ns | >0.9999 |
| **wt** | **0.01057** | **0.006928** |  |  | **0.1381** | **0.07713** |  |  | **0.339** | **0.1058** |  |  |

**Table 11.** One-way ANOVA test of enzymatic activity of **Chitinase** overtime produced by recovered strains grown in **CCMM** compared to the wild type. P values: 0.1234 (ns), 0.0332 (*), 0.0021 (**), 0.0002 (***), <0.0001 (****).

| **Strain** | **0 days** | | | | **2 days** | | | | **4 days** | | | |
| --- | --- | --- | --- | --- | --- | --- | --- | --- | --- | --- | --- | --- |
|  | **Mean** | **StDev** | **Sig** | **P Adj** | **Mean** | **StDev** | **Sig** | **P Adj** | **Mean** | **StDev** | **Sig** | **P Adj** |
| MC1 | 0.01417 | 0.003147 | ns | >0.9999 | 2.01 | 2.271 | ns | 0.9957 | 2.747 | 3.053 | ns | 0.9894 |
| MC2 | 0.007791 | 0.000625 | ns | >0.9999 | 0.05649 | 0.01603 | ns | 0.9999 | 0.1701 | 0.07244 | ns | 0.9995 |
| MC3 | 0.008795 | 0.007188 | ns | >0.9999 | 0.03382 | 0.002321 | ns | 0.9998 | 0.07951 | 0.01284 | ns | 0.9994 |
| MC4 | 0.01098 | 0.003443 | ns | >0.9999 | 0.07437 | 0.002471 | ns | 0.9999 | 0.1908 | 0.01205 | ns | 0.9995 |
| MC5 | 0.0317 | 0.00078 | ns | 0.9998 | 0.1189 | 0.05886 | ns | 0.9999 | 0.4459 | 0.17 | ns | 0.9997 |
| MC6 | 0.1391 | 0.05132 | ns | 0.999 | 5.108 | 4.769 | ns | 0.2945 | 7.205 | 5.499 | * | 0.0257 |
| MC7 | 0.5938 | 0.4122 | ns | 0.0582 | 0.07124 | 0.03735 | ns | 0.9999 | 0.1084 | 0.1001 | ns | 0.9994 |
| MG1 | 0.7642 | 0.4441 | ** | 0.0051 | 8.488 | 6.233 | ** | 0.0057 | 13.28 | 0.8293 | **** | <0.0001 |
| MG2 | 1.108 | 0.15 | **** | <0.0001 | 6.406 | 1.874 | ns | 0.0825 | 9.795 | 1.611 | *** | 0.0004 |
| MG3 | 0.7509 | 0.4001 | ** | 0.0063 | 7.409 | 8.096 | * | 0.0248 | 7.462 | 7.636 | * | 0.0176 |
| MG4 | 2.787 | 0.5707 | **** | <0.0001 | 8.415 | 0.2703 | ** | 0.0063 | 10.87 | 0.4333 | **** | <0.0001 |
| MG5 | 1.747 | 0.6252 | **** | <0.0001 | 7.257 | 3.116 | * | 0.0301 | 7.03 | 1.167 | * | 0.0329 |
| MG6 | 0.7196 | 0.1777 | * | 0.0102 | 6.276 | 1.942 | ns | 0.0952 | 8.751 | 2.375 | ** | 0.0022 |
| MG7 | 0.007496 | 0.004844 | ns | >0.9999 | 0.6911 | 0.7777 | ns | 0.9998 | 0.9831 | 1.065 | ns | >0.9999 |
| MG8 | 0.006965 | 0.000668 | ns | >0.9999 | 0.04297 | 0.01264 | ns | 0.9999 | 0.2388 | 0.2052 | ns | 0.9995 |
| MG9 | 0.02013 | 0.000297 | ns | >0.9999 | 0.3116 | 0.3025 | ns | >0.9999 | 0.5651 | 0.464 | ns | 0.9997 |
| MG10 | 0.01068 | 0.002749 | ns | >0.9999 | 0.1777 | 0.07444 | ns | >0.9999 | 0.6048 | 0.1374 | ns | 0.9998 |
| MG11 | 0.01133 | 0.00118 | ns | >0.9999 | 0.005017 | 0.00312 | ns | 0.9998 | 0.05784 | 0.009108 | ns | 0.9994 |
| MG12 | 0.6052 | 0.6906 | ns | 0.0503 | 0.3164 | 0.09108 | ns | >0.9999 | 2.272 | 2.505 | ns | 0.999 |
| MG13 | 0.01393 | 0.00137 | ns | >0.9999 | 0.01971 | 0.01412 | ns | 0.9998 | 0.05566 | 0.02873 | ns | 0.9994 |
| **wt** | **0.003306** | **0.003847** |  |  | **0.324** | **0.1304** |  |  | **0.9323** | **0.3179** |  |  |

**Table 12.** One-way ANOVA test of enzymatic activity of **Glucanases** overtime produced by recovered strains grown in **CCMM** compared to the wild type. P values: 0.1234 (ns), 0.0332 (*), 0.0021 (**), 0.0002 (***), <0.0001 (****).

| **Strain** | **0 days** | | | | **2 days** | | | | **4 days** | | | |
| --- | --- | --- | --- | --- | --- | --- | --- | --- | --- | --- | --- | --- |
|  | **Mean** | **StDev** | **Sig** | **P Adj** | **Mean** | **StDev** | **Sig** | **P Adj** | **Mean** | **StDev** | **Sig** | **P Adj** |
| MC1 | 0.1397 | 0.02768 | ns | 0.9997 | 3.859 | 3.271 | ns | 0.9874 | 7.055 | 4.26 | ns | 0.9994 |
| MC2 | 0.09444 | 0.005309 | ns | 0.9997 | 1.238 | 0.1337 | ns | 0.5664 | 20.28 | 27.51 | ns | 0.9089 |
| MC3 | 0.3089 | 0.2223 | ns | >0.9999 | 1.699 | 1.124 | ns | 0.6745 | 3.425 | 2.059 | ns | 0.9942 |
| MC4 | 0.1444 | 0.04082 | ns | 0.9997 | 2.759 | 1.979 | ns | 0.8921 | 6.874 | 4.222 | ns | 0.9994 |
| MC5 | 0.902 | 0.2132 | ns | 0.9945 | 2.904 | 1.171 | ns | 0.9137 | 7.658 | 3.677 | ns | 0.9996 |
| MC6 | 1.253 | 0.4925 | ns | 0.8676 | 8.061 | 2.481 | ns | 0.9997 | 14.52 | 0.8636 | ns | 0.9992 |
| MC7 | 1.298 | 0.4943 | ns | 0.8285 | 0.7049 | 0.1916 | ns | 0.448 | 3.889 | 2.209 | ns | 0.9949 |
| MG1 | 1.538 | 0.6292 | ns | 0.5757 | 11.96 | 4.569 | ns | 0.8254 | 23.61 | 6.797 | ns | 0.6106 |
| MG2 | 2.124 | 0.4047 | ns | 0.1278 | 14.06 | 4.358 | ns | 0.3643 | 21.26 | 4.289 | ns | 0.8366 |
| MG3 | 1.779 | 1.076 | ns | 0.3389 | 17.17 | 13.54 | * | 0.0473 | 30.31 | 24.1 | ns | 0.1274 |
| MG4 | 8.353 | 2.276 | **** | <0.0001 | 26.81 | 5.484 | **** | <0.0001 | 37.12 | 7.351 | * | 0.0123 |
| MG5 | 1.841 | 0.4018 | ns | 0.2896 | 9.492 | 1.367 | ns | 0.999 | 13.84 | 1.661 | ns | 0.9994 |
| MG6 | 1.225 | 0.4695 | ns | 0.8893 | 10.39 | 2.332 | ns | 0.9884 | 16.42 | 1.461 | ns | 0.9954 |
| MG7 | 0.07349 | 0.03768 | ns | 0.9996 | 4.922 | 5.192 | ns | 0.999 | 10.12 | 6.858 | ns | >0.9999 |
| MG8 | 0.0252 | 0.006533 | ns | 0.9995 | 1.458 | 0.5166 | ns | 0.6179 | 19.66 | 22.89 | ns | 0.9423 |
| MG9 | 0.3493 | 0.0149 | ns | 0.9999 | 1.296 | 1.075 | ns | 0.5801 | 7.391 | 1.305 | ns | 0.9995 |
| MG10 | 0.1032 | 0.01555 | ns | 0.9997 | 2.864 | 0.22 | ns | 0.908 | 9.848 | 1.376 | ns | >0.9999 |
| MG11 | 0.2363 | 0.02512 | ns | >0.9999 | 0.245 | 0.1621 | ns | 0.357 | 5.105 | 1.774 | ns | 0.999 |
| MG12 | 2.214 | 2.369 | ns | 0.0955 | 6.401 | 1.614 | ns | 0.9997 | 13.03 | 4.15 | ns | 0.9996 |
| MG13 | 0.08936 | 0.01008 | ns | 0.9997 | 0.2628 | 0.2204 | ns | 0.3601 | 2.309 | 0.8306 | ns | 0.9847 |
| **wt** | **0.2792** | **0.2182** |  |  | **7.173** | **7.083** |  |  | **10.34** | **9.683** |  |  |


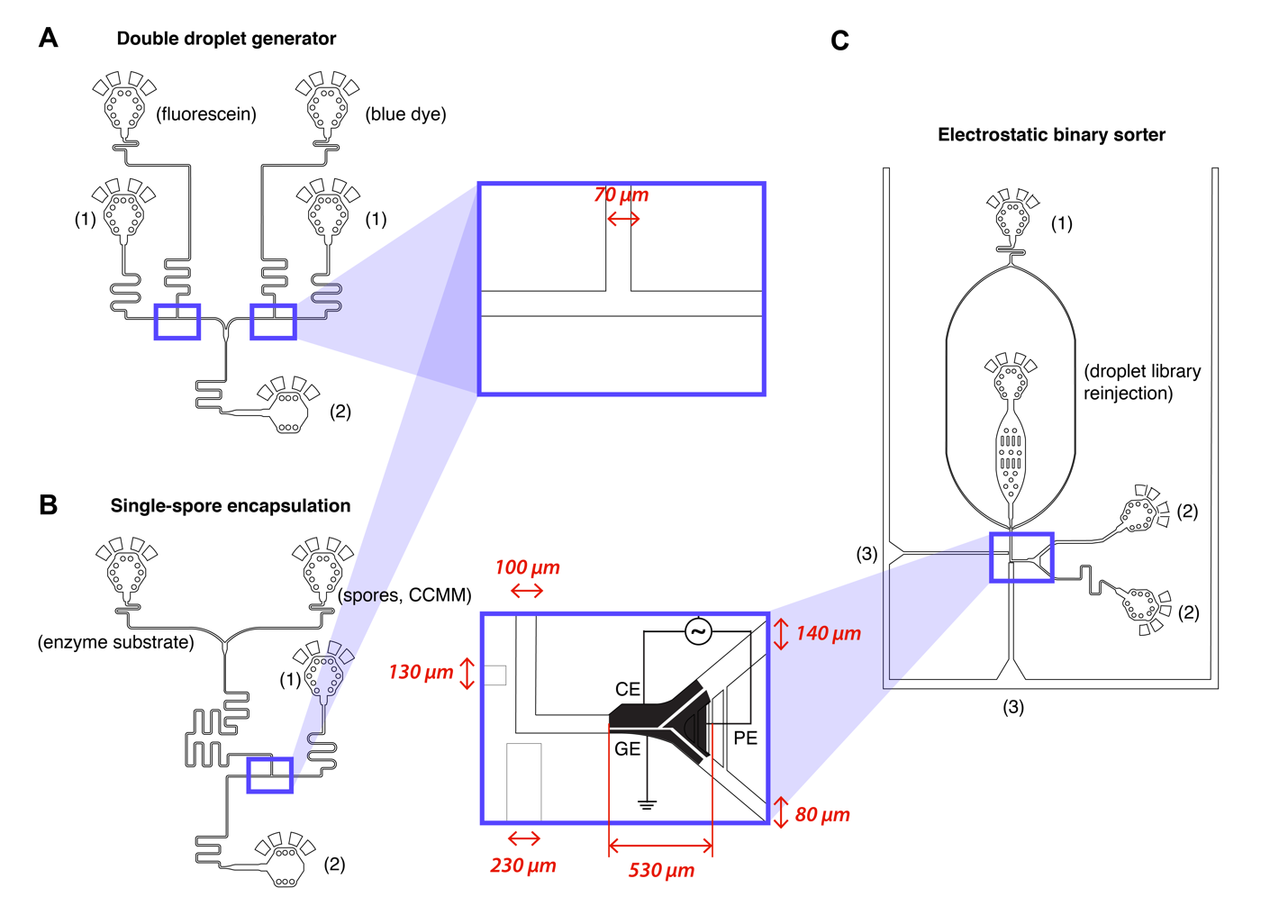


**Figure 1 - Microfluidic devices for (A) optimizing sorting parameters and (B and C) fungal enzyme screening.** Two T-junction droplet generators (left) were designed with 70 x 70 (w x h) µm channel dimensions. (A) A first droplet generator was used for creating a mixed droplet library for sorter characterization**.** Fluorescein and blue dye droplets were generated with two separately controlled T-junction droplet generators that produced droplets of the same size (oil/aqueous flow ratio identical) at different flow rates. (B) A second droplet generator was used to generate a filamentous fungi droplet library. An enzymatic substrate and a spore solution were injected at same flow rates (~ 100 nL•s^-1^), and mixed through a serpentine mixer before droplet generation. Droplets were collected from the outlet (labeled as (2)) into a PCR tube. For both devices, the fluorinated oil (HFE 7500 2% fluoro-based surfactant) was injected into the inlet (labeled as (1)). (C) The microfluidic binary sorter was designed with co-planar electrodes under a dielectric (fabrication similar to our previously reported work^1,2^). The emission fiber channel (3) has a 100 µm height for the excitation fiber and a 170 µm height for the emission fiber. All other dimensions are as indicated on the zoomed-in sorting region. The droplet library was re-injected into the device inlet and droplets were spaced with spacer oil (1). After sorting, droplets were collected from the outlets (2).

***
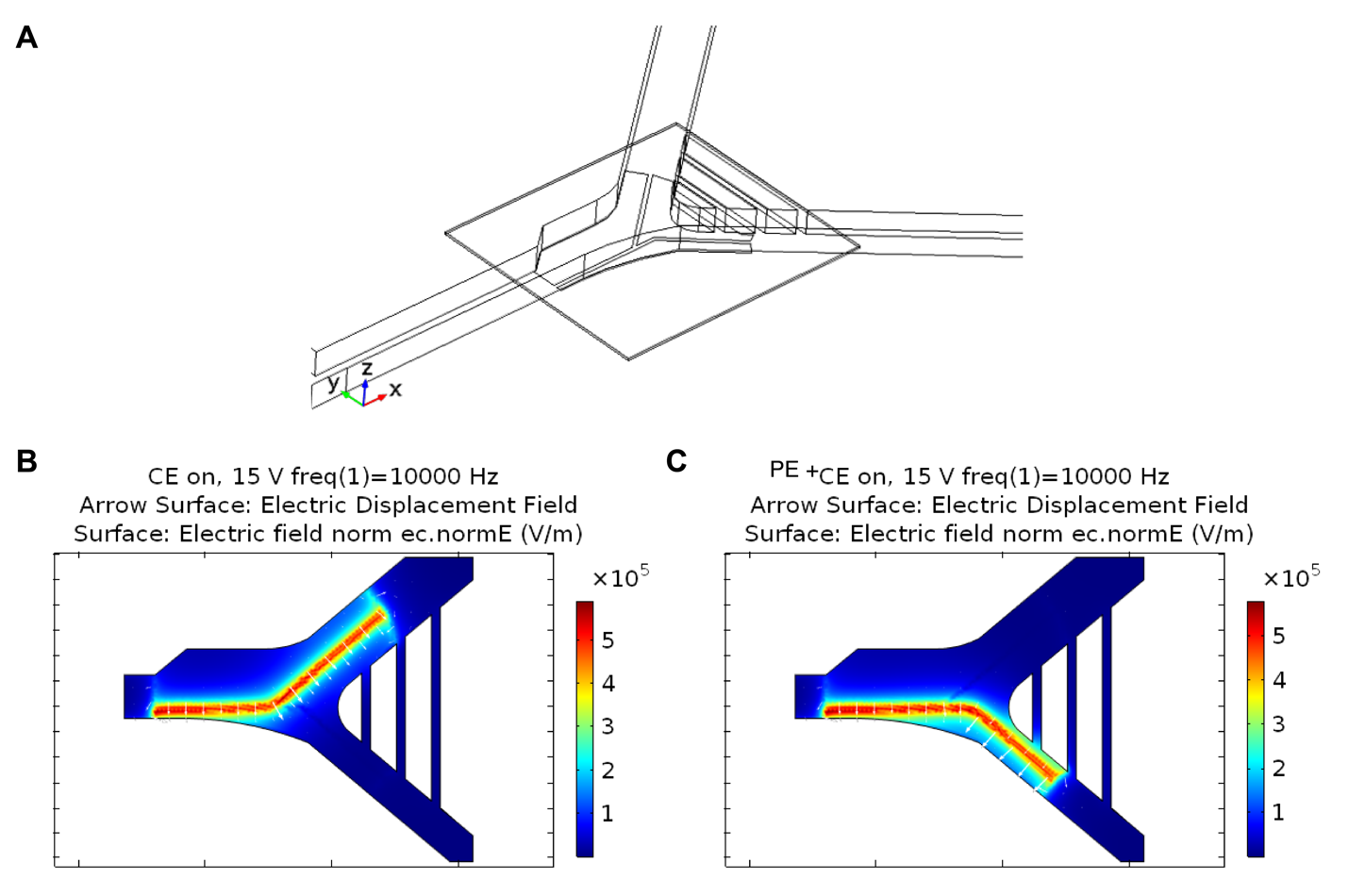
***

**Figure 2 - Simulation of the electric field above the electrodes.** (A) Model components include a channel layer with PDMS boundary and HFE 7500 domain, a 7 µm thick dielectric layer and three electrode terminals. (B) Top view of electrostatic displacement field vectors and electric field strength (V/m) at z = 7 µm (SU-8 5 dielectric with HFE7500 oil interface) when the constant electrode (CE) is applied with 15 V_RMS_ at 10 kHz. The electrostatic force vectors are perpendicular to the gap and to the flow. (C) Top view of electrostatic field strength at z = 7 µm when both the CE and the pulsing electrode (PE) are on with 15 V_RMS_ applied.


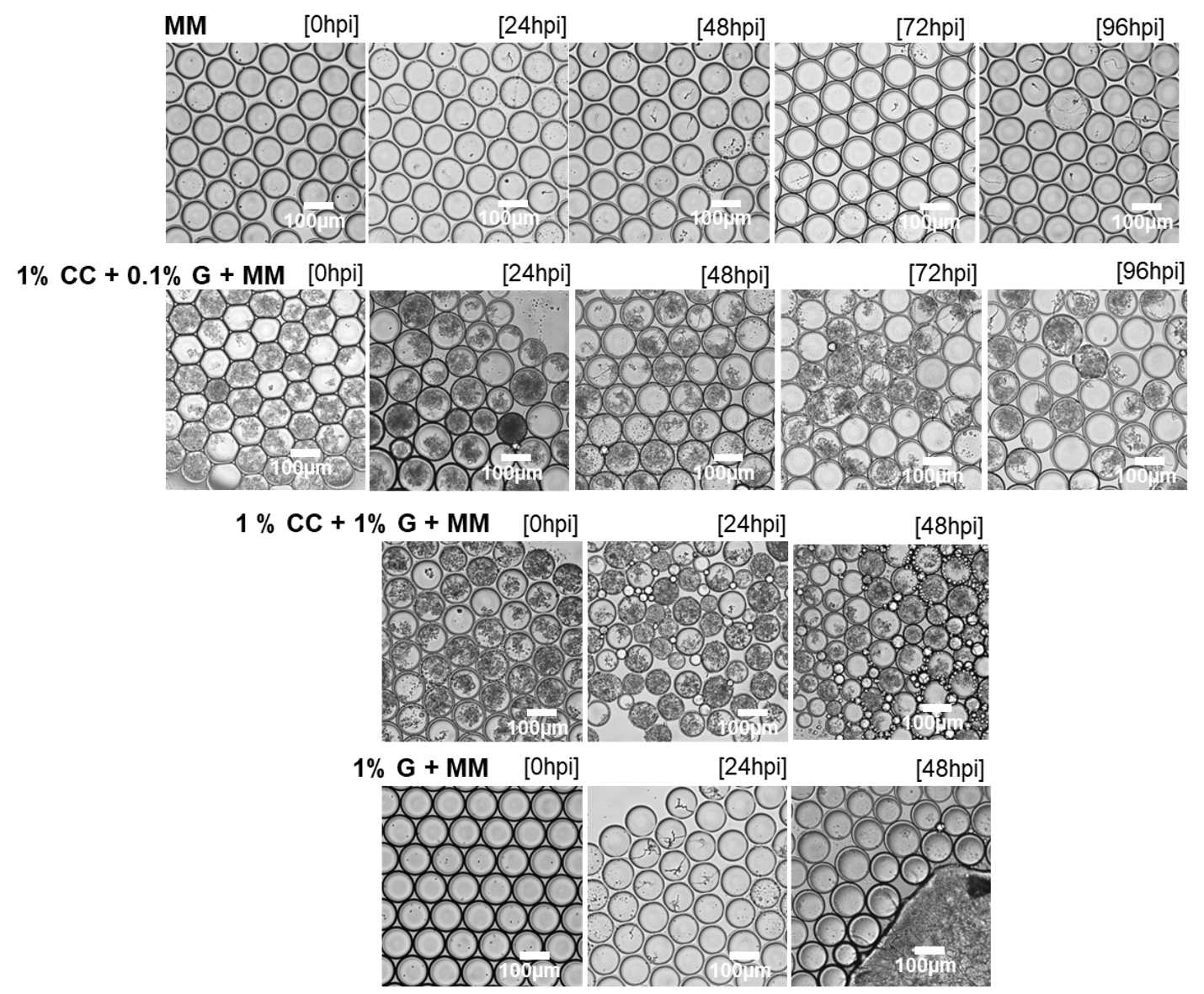


**Figure 3 - Incubation of filamentous fungi in the droplets with varying media**.  Bright field microscopy images of 1 nL droplets incubated in oil with 2 % fluorosurfactant for 96 hours (40x) in a PCR tube. In droplets containing colloidal chitin (CC), the filamentous fungi show hyphal growth around the colloidal chitin and minimizes the hyphae to exit the droplets. After 48 hpi, many of the hyphae in 1% G burst the droplet, causing many of the droplets to merge. Droplets containing 0.1% G or minimal media shows low rates of hyphal exiting, enabling droplets being intact for > 4 days.


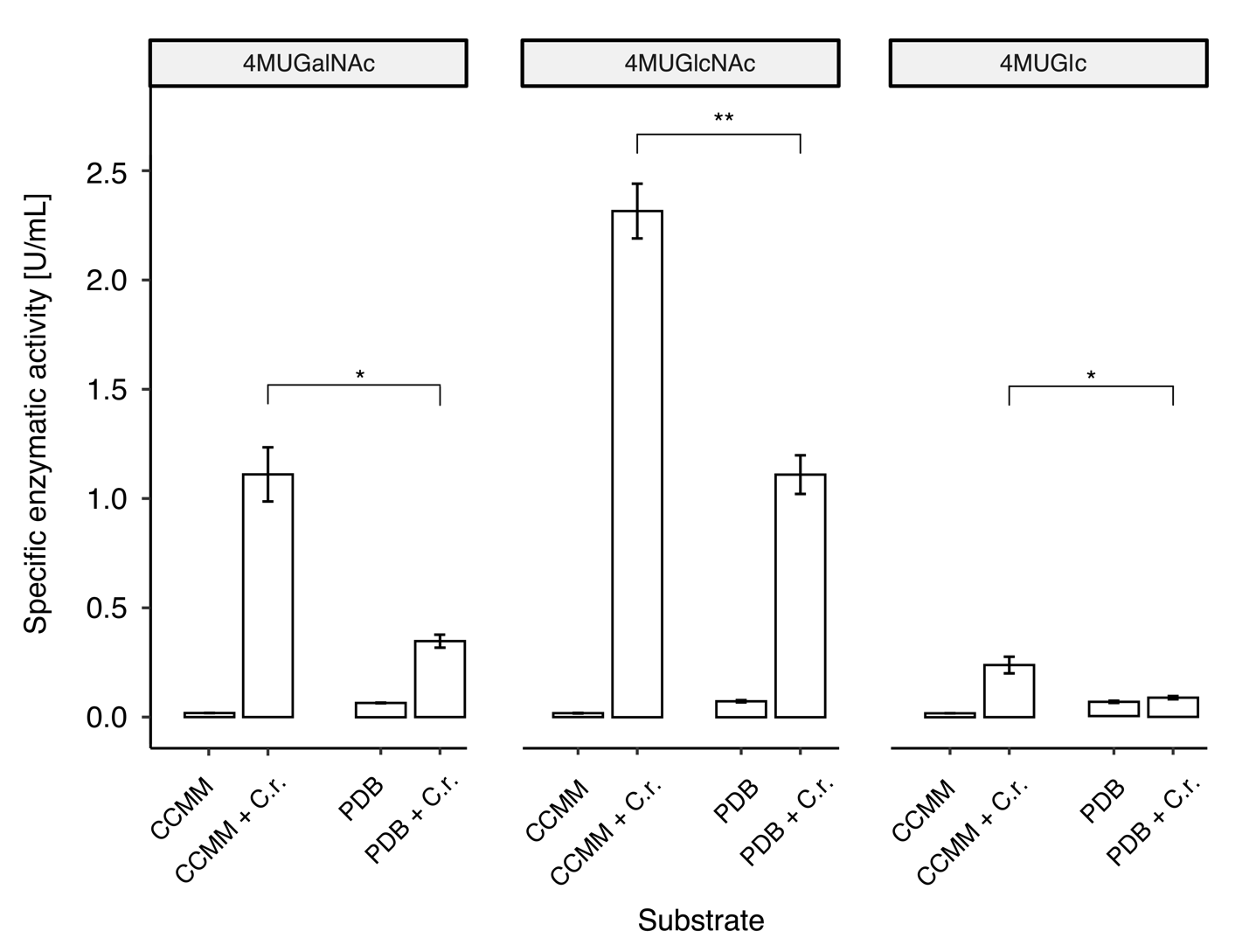


**Figure 4 - *C.rosea* well plate end-point enzymatic assay.** The enzymatic activity of cell wall degrading enzymes in *C. rosea* cultures (27 ºC, 5 days, 200 mL) was compared in different media formulations (colloidal chitin minimal media, CCMM, and potato dextrose broth, PDB). Enzymatic activity is displayed as a specific enzymatic activity (U/mL) using 4-MU-GlcNAc, 4-MU-GalNAc, and 4-MU-Glc as substrates for chitinase, N-acetyl galactosaminidase, and glucanase activity, respectively. A unpaired Welch t-test was performed between the enzymatic activity in *C.rosea* grown in solid and liquid media (* for P < 0.05, ** for P < 0.01) (30 min, 37 ºC, pH 5.1). Error bars represent one standard deviation, N = 3.

***
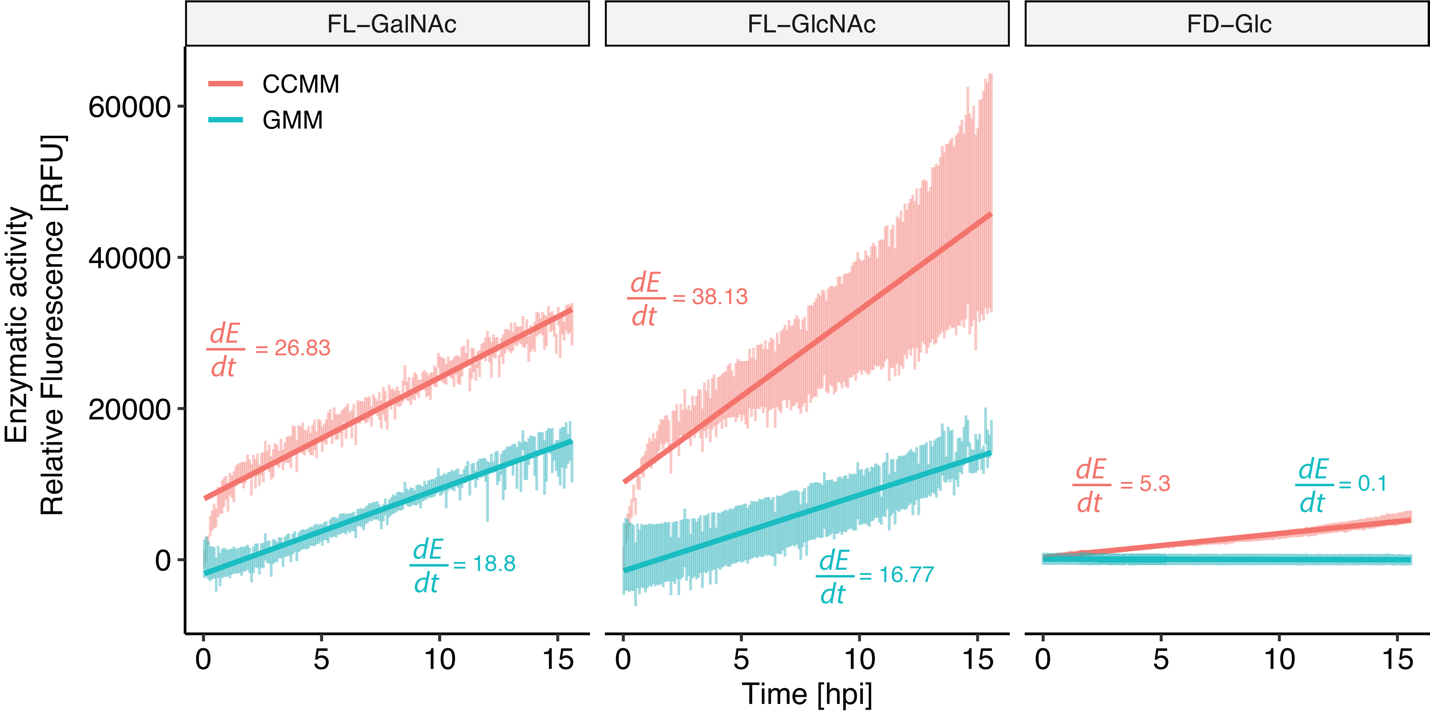
***

**Figure 5 - *C.rosea* conidia well-plate kinetic assay using fluorescein-based substrates.** The enzymatic activity of cell wall degrading enzymes in *C. rosea* cultures were compared between different media (colloidal chitin minimal media, CCMM (red) and glucose minimal media, GMM (blue)) over 15 h post-incubation. Germination started at 0 h from parent strain spores at concentrations as in droplets (0.5 x10^7^ spores•mL^-1^) and were incubated (27 ºC, 16 h) with either FL-GlcNAc, FL-GalNAc or FD-Glu as the substrate. At each measured time point, one standard deviation is shown, N = 3. Relative fluorescence is normalized with a control (media and substrate without spores) and slopes dE/dt represent change in enzymatic activity over time. As shown, colloidal chitin media offers a faster increase in fluorescence rate, which will allow for shorter incubation times in droplets.

***
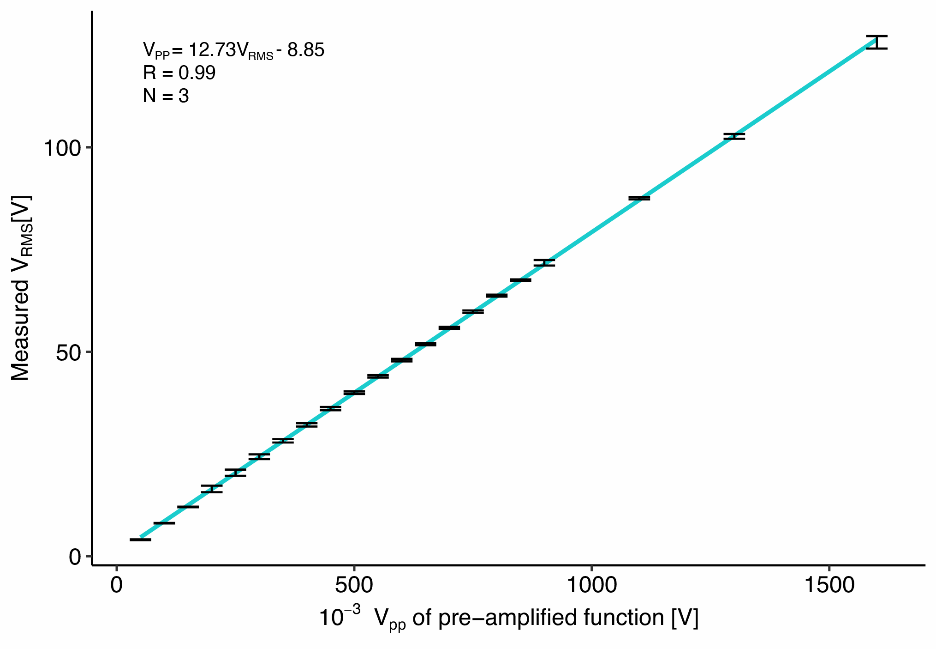
***

**Figure 6 - Amplifier calibration curve.** Measured root-mean squared voltage at the electrode, after amplification of a sine wave (10 kHz, variable V_PP_) using a PZD-700A, Trek Inc. amplifier. The gain at 10 kHz was determined to be 78.85. The resulting linear relationship was used to calculate the true applied potential (V_RMS_) in experiments and simulations.


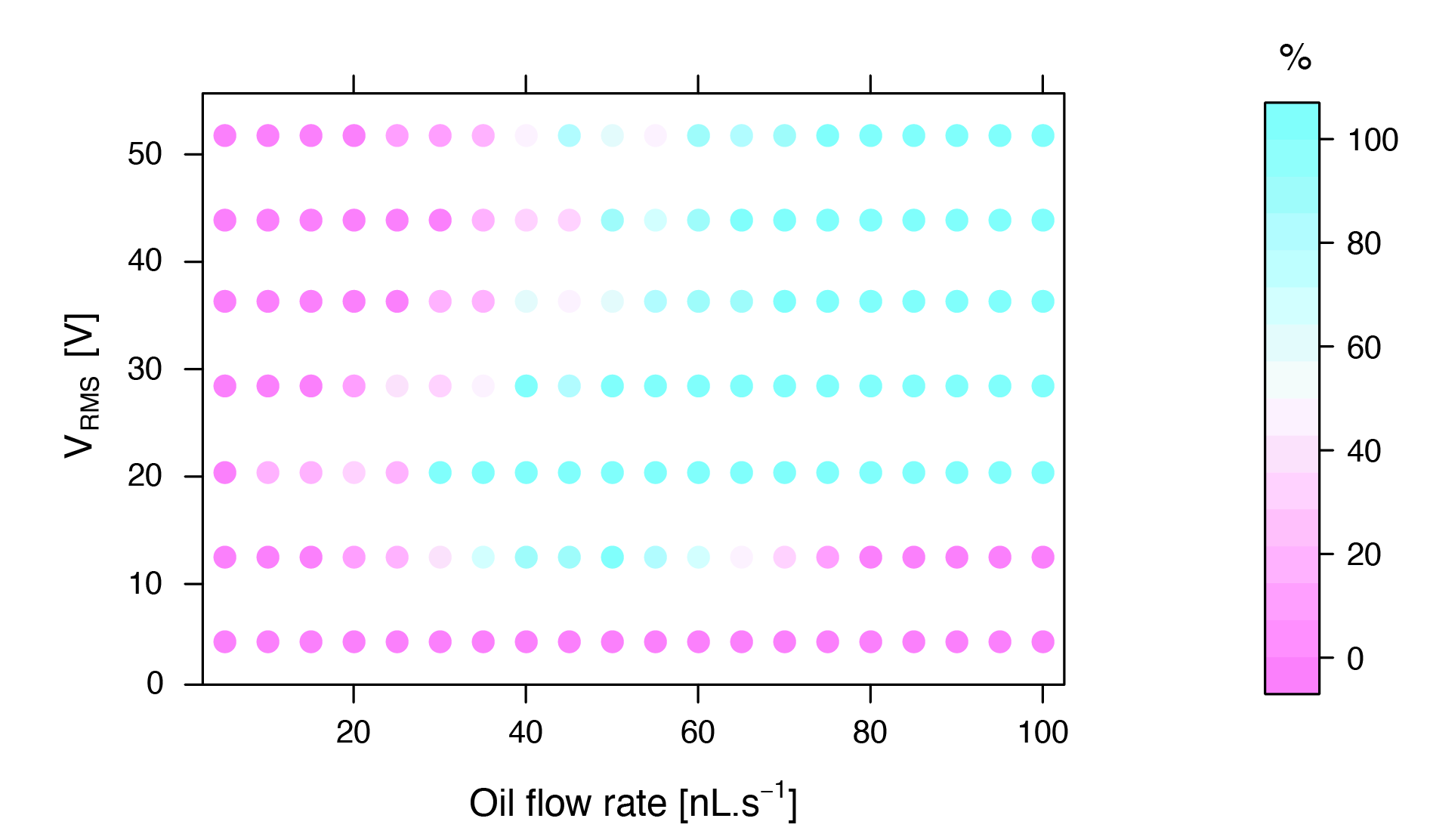


**Figure 7 - Efficiency of sorting under different flow rates and applied potential.** Dot plot showing the success rate of sorting ([# successfully sorted droplets (T) / # of total droplets sorted (T+F)]* 100 %) in a 7 x 20 factorial design experiment with binary outcome (N = 10). Each dot (or condition), the potential applied to the sorting electrodes were kept at constant frequency (10 kHz), while the spacer oil flowrate was varied. Droplets were generated at 0.5 nL•s^-1^ (water) and 1 nL•s^-1^ (oil and surfactant). Blue dots indicate sorting conditions with 100 % sorting success.


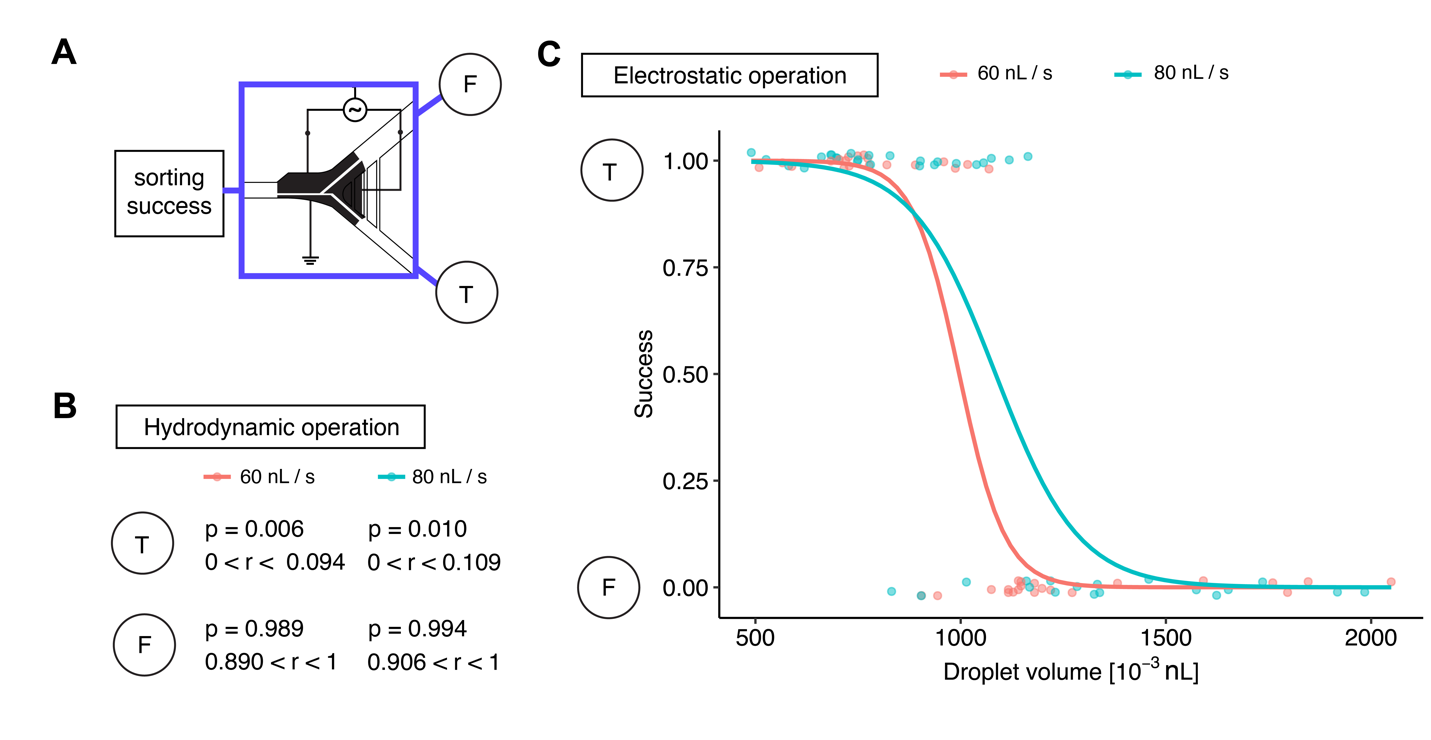


**Figure 8 – Sorting success for a polydisperse volume** **droplet library**.  (A) Droplets were sorted using a co-planar sorter. A “positive” droplet going into the main channel was considered a sorting failure (F), while a droplet going into the disfavored channel was considered a sorting success (T) (see sorting design schematic). (B) Sorting logic outcome under hydrodynamic operation (free flow without the use of electrodes). The probability of polydisperse volume droplets to enter the disfavored channel was measured under two flow conditions (60 nL•s^-1^ spacer oil; 80 nL•s^-1^ spacer oil). At 60 nL•s^-1^, the probability p of sorting failure due to a false positive (droplet entering the disfavored channel, T, without actuation) is ~0.6%.  At 80 nL•s^-1^, the sorting failure probability is ~1%.  (C) Sorting logic outcome under electrostatic sorting.  Sorting success of polydisperse volume droplet libraries were measured under two electrostatic sorting regimes (36.3 V_RMS_ actuation, 60 nL•s^-1^ spacer oil; 44.2 V_RMS_ actuation, 80 nL•s^-1^ spacer oil).  Logic outcome was fitted with a logarithmic binomial regression fit.  On average, droplets smaller than ~ 1 nL can be sorted with near-perfect efficiency.

***
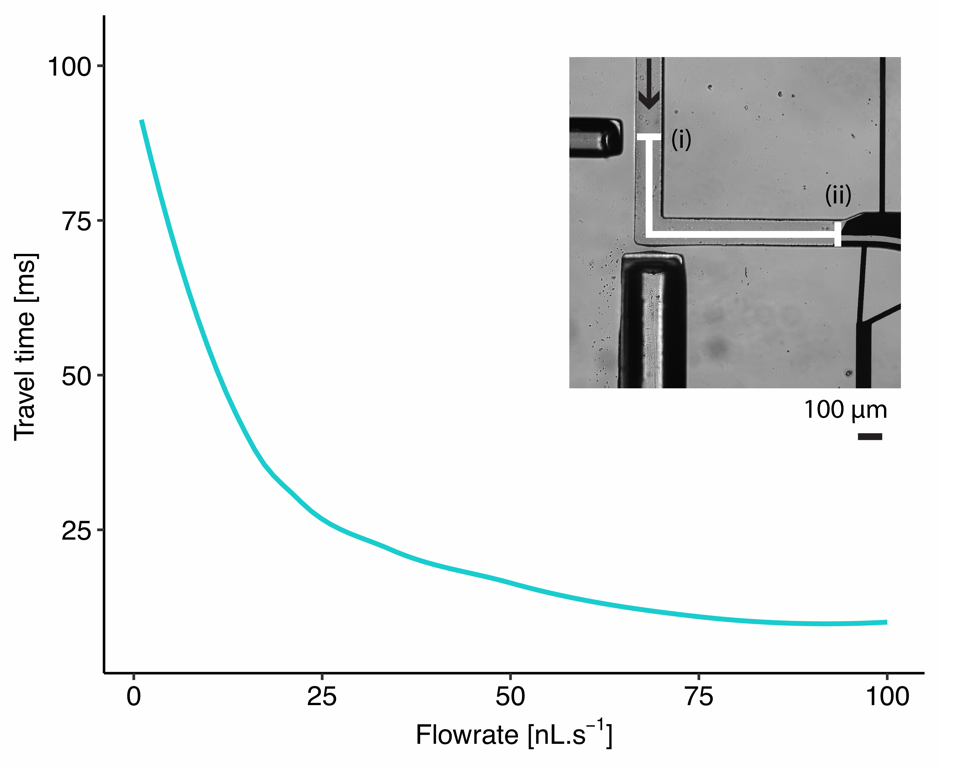
***

**Figure 9 - Droplet sorting timing calibration.** Droplet travel time from detection point (i) to sorting point (ii) (~ 1.1 mm) under different oil flow rates. Droplet generation was kept at 3 nL•s^-1^ aqueous (ddH_2_O) and 4 nL•s^-1^ oil phase (HFE 7500 0.5% fluorosurfactant), while the spacer oil phase varied in flow rate. Time was measured by summing up exposure times of frames in high-speed image series (each frame was captured every ~33 ms).
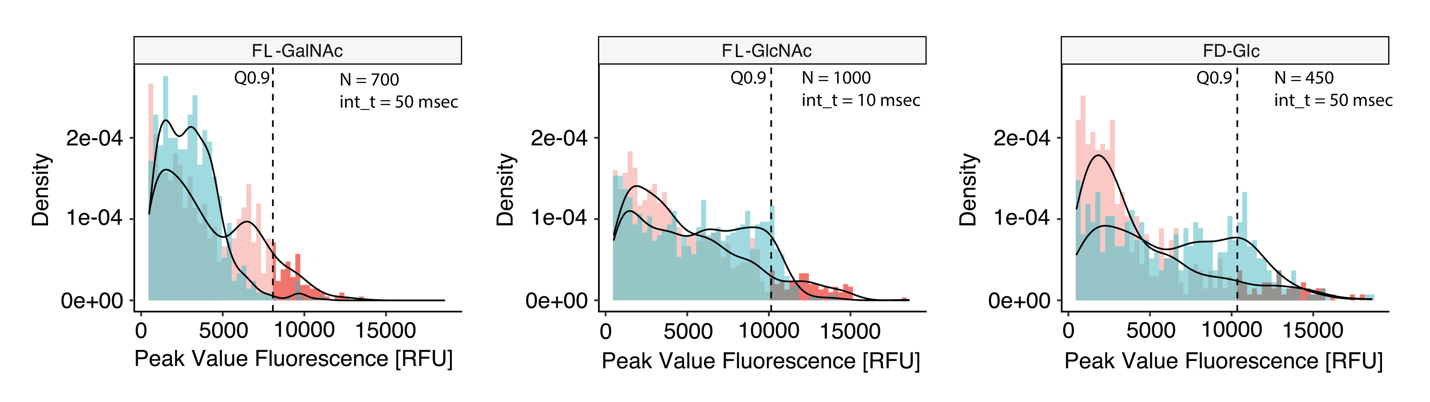


**Figure 10 - Sorting histograms of single-spore droplet libraries for each fluorescein substrate.** Fluorescence intensity of each droplet in a mutant population (shown as red) and a wild type (shown as blue) population incubated at 27 ºC before microfluidic sorting. Intensity of peaks between the wavelengths 510 nm and 520 nm were used for gating. To sort droplets, the intensity gate was set at the 0.9 quantile of the mutant population fluorescence (shown by the dotted line).


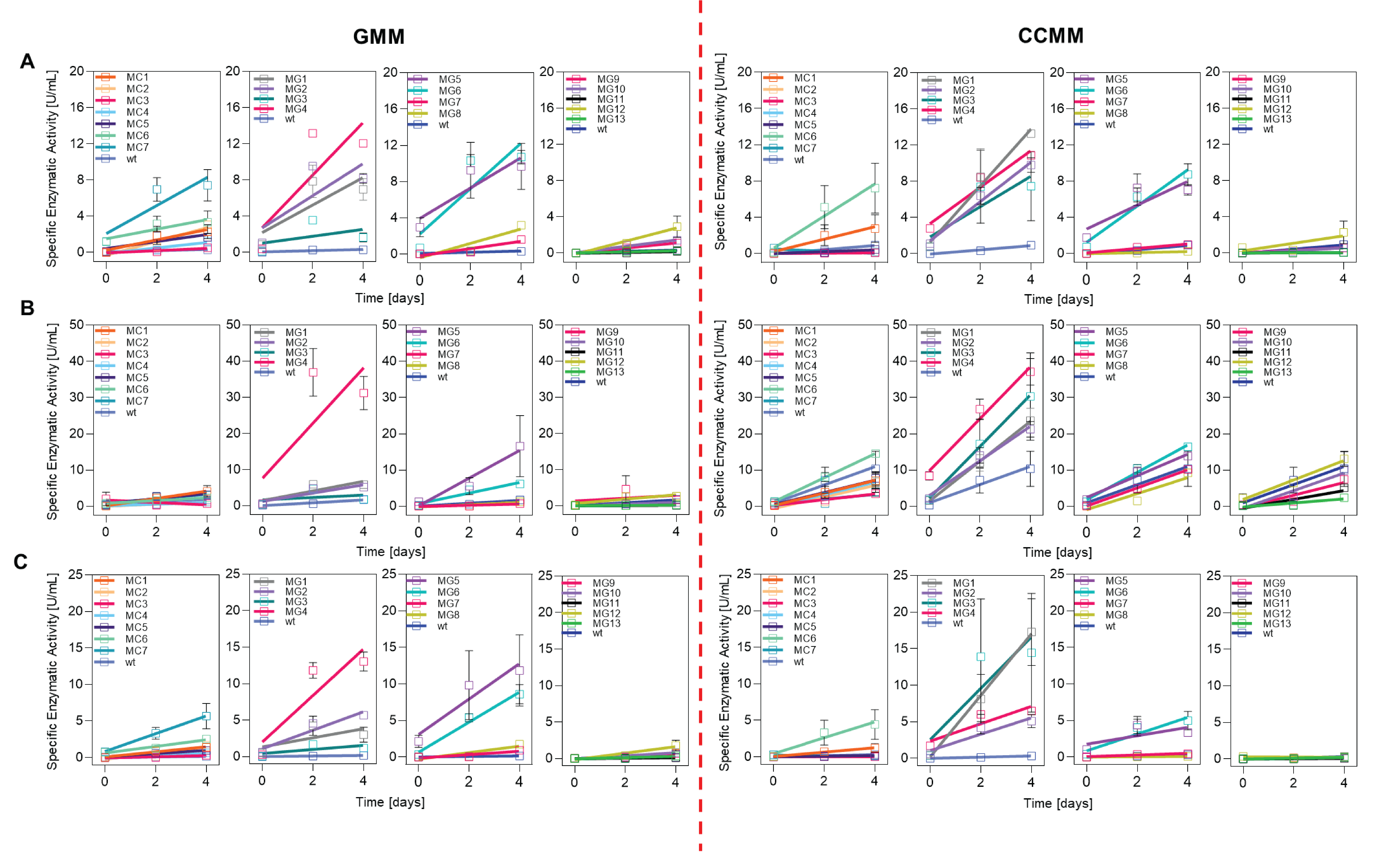


**Figure 11 - Production of cell-wall degrading enzymes by strains recovered from the microfluidic screening.** Third generation strains were incubated over 4 days in CCMM and GMM liquid culture media and specific enzymatic activity [U/mL] values were obtained through an end-point enzymatic assay (pH 5.1, 30 min) (N = 4 for each point). Plots represent the enzymatic activity of (A) chitinase (B) glucopyranosidase, and (C) N-acetylgalactosaminidase cultured in 1 % colloidal chitin and minimal media (CCMM; left) and 1 % glucose and minimal media (GMM; right). Error bars represent one standard error.


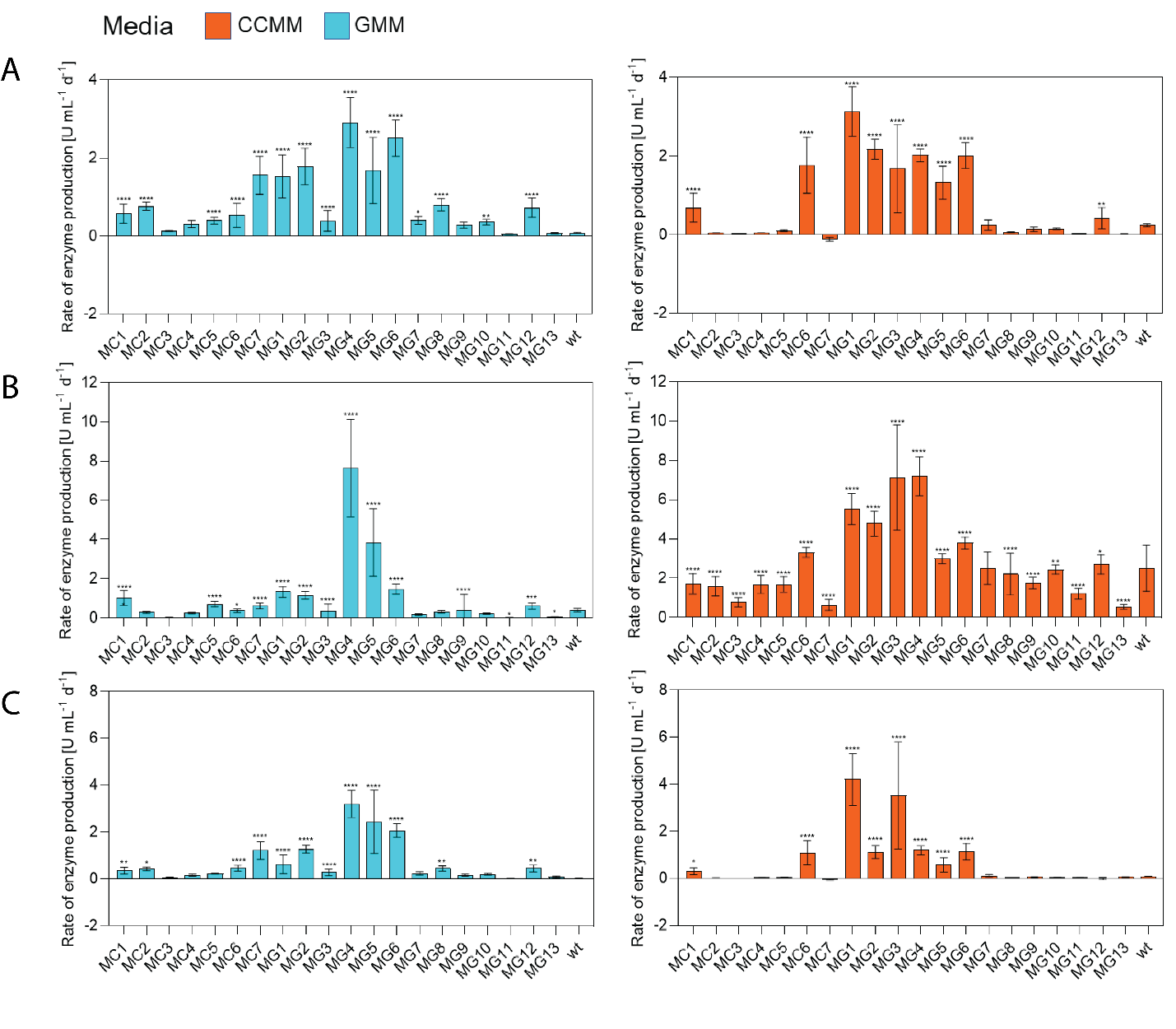


**Figure 12 – Rate of enzymatic production of strains recovered from the microfluidic screening after three generations of culture.** Plots of the rates for the production (A) chitinase, (B) glucopyranosidase and (C) N-acetylgalactosaminidase were obtained from the slope of simple linear regression, N = 4 (see Figure S11). Error bars representing standard error, one-way ANOVA against *WT*, P values: 0.1234 (ns), 0.0332 (*), 0.0021 (**), 0.0002 (***), <0.0001 (****).

***
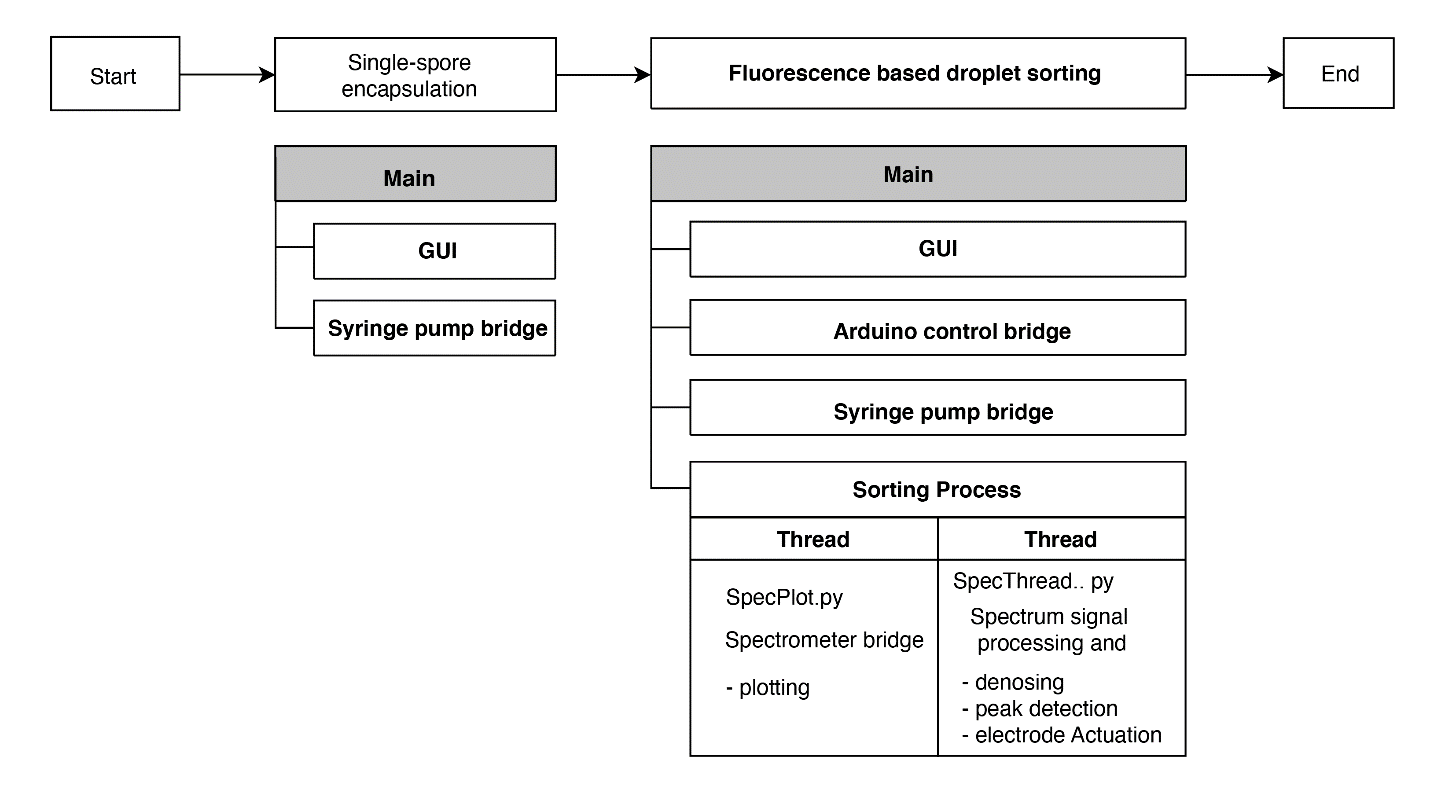
***

**Figure 13 - Software workflow.** The Python 3.9 based software is used for two independent experimental steps: single-spore encapsulation and fluorescence-based droplet sorting. For each experiment, a main python process is started to drive hardware and provide a user interface. For single-cell encapsulation, the main process runs a GUI process and a syringe pump bridge process, controlling the pressure driven syringe pumps. For fluorescence-based droplet sorting, the main process executes a GUI process, an Arduino control bridge which operates switches supplying high-voltage to the electrodes, a syringe pump process, and a sorting process. The sorting process contains two sub-processes: a bridge with the spectrometer instrument continuously reading raw intensities and wavelengths, and a signal processing process for signal denoising, background subtraction, peak detection and electrode actuation. Software can be found under GNU GPL v3.0 on <https://bitbucket.org/shihmicrolab/fungalmicrofluidics/>.


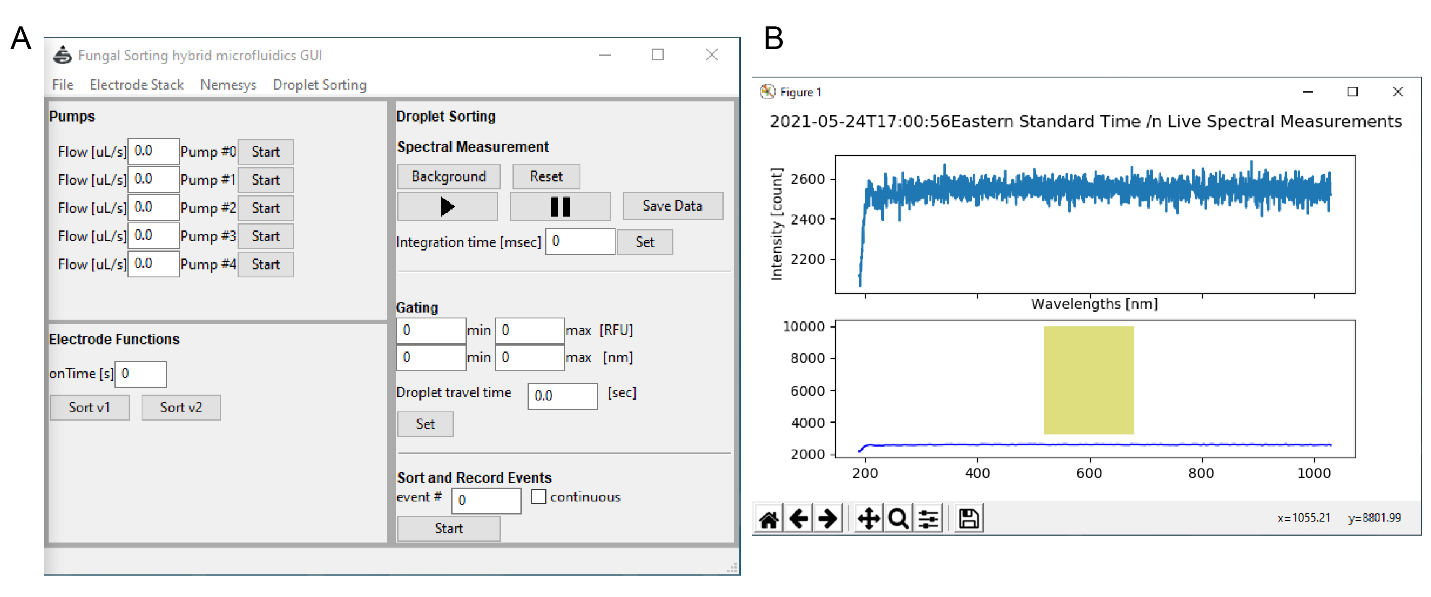


**Figure 14 - Graphical user interface for sorting.** (A) An image of the control panel graphical user interface for fungal screening. After running a main script, the GUI can be used to control pumps, the electrode switching board, and the spectrometer. The menu ribbon can be used to control the hardware interface and to start and to stop communication with the hardware. Pumps can be controlled in the pump panel by configuring the flow rates of the respective pump and using the start button to start and stop fluid dispensing. Electrodes can be controlled in the electrode panel. Electrode ‘onTime’ can be inputted and buttons are present for users to program custom actuation sequences (e.g., Sort v1 and Sort v2 are pre-made sequences for sorting). The Droplet Sorting panel is used to perform automated droplet sorting. A spectrum viewer can be controlled using the play buttons, data snapshots can be saved, spectrometer integration time can be set, and background fluorescence can be subtracted using the top panel. These operations can be applied to “live” measurements. In the middle panel, the sorting gate (wavelength and intensity) and droplet travel time can be set. The user can start the autonomous sorting by performing a continuous measurement or a selected number of events. The procedure can be paused, and events can be saved in a data file. (B) Spectrum viewer graphical user interface. Raw spectrum (blue) of the emission fiber transferred signal, showing the wavelength and measured intensities (RFU), measured by the spectrometer (top). The spectrum after signal processing (bottom) is also displayed through live plotting. The plotted signal (blue) has the background noise subtracted and is denoised using a third order Butterworth lowpass filter. The user sets the sorting gate and is displayed (shown as a green box).
